# A circRNA–mRNA pairing mechanism regulates tumor growth and endocrine therapy resistance in ER-positive breast cancer

**DOI:** 10.1073/pnas.2420383122

**Published:** 2025-02-18

**Authors:** Jia Yi, Jiao Du, Xue Chen, Rui-chao Nie, Guo-sheng Hu, Lei Wang, Yue-ying Zhang, Shang Chen, Xiao-sha Wen, Di-xian Luo, Hua He, Wen Liu

**Affiliations:** ^a^State Key Laboratory of Cellular Stress Biology, School of Pharmaceutical Sciences, Xiamen University, Xiamen 361102, Fujian, China; ^b^Fujian Provincial Key Laboratory of Innovative Drug Target Research, School of Pharmaceutical Sciences, Xiamen University, Xiamen 361102, Fujian, China; ^c^Xiang An Biomedicine Laboratory, School of Pharmaceutical Sciences, Xiamen University, Xiamen 361102, Fujian, China; ^d^Yu-Yue Pathology Scientific Research Center, Chongqing 400039, China; ^e^Jinfeng Laboratory, Chongqing 401329, China; ^f^National Institute for Data Science in Health and Medicine, Xiamen University, Xiamen 361102, Fujian, China; ^g^Laboratory Medicine Centre, Shenzhen Nanshan People’s Hospital, Shenzhen 518052, Guangdong, China; ^h^The Third Affiliated Hospital (Luohu Hospital), Shenzhen University, Shenzhen 518000, Guangdong, China; ^i^Department of Neurosurgery, Third Affiliated Hospital, Naval Medical University, Shanghai 200438, China

**Keywords:** breast cancer, endocrine therapy resistance, circFOXK2, CCND1, RNA-RNA pairing

## Abstract

The molecular mechanisms underlying estrogen receptor (ER)-positive breast carcinogenesis and drug resistance remain incompletely understood. Elevated expression of CCND1 is linked to enhanced invasiveness, poorer prognosis, and resistance to drug therapies in ER-positive breast cancer. In this study, we identify a highly expressed circular RNA (circRNA) derived from *FOXK2*, called circFOXK2, which plays a key role in stabilizing *CCND1* mRNA, thereby promoting cell cycle progression, cell growth, and endocrine therapy resistance in ER-positive breast cancer cells. Mechanistically, circFOXK2 binds directly to *CCND1* mRNA via RNA–RNA pairing and recruits the RNA-binding protein ELAVL1/HuR, stabilizing the *CCND1* mRNA and enhancing CCND1 protein levels. This results in activation of the CCND1–CDK4/6–p-RB–E2F signaling axis, driving the transcription of downstream E2F target genes and facilitating the G1/S transition during cell cycle progression. Notably, targeting circFOXK2 with antisense oligonucleotide (ASO-circFOXK2) suppresses ER-positive breast cancer cell growth both in vitro and in vivo. Moreover, combination therapy with ASO-circFOXK2 and tamoxifen exhibits synergistic effects and restores tamoxifen sensitivity in tamoxifen-resistant cells. Clinically, high circFOXK2 expression is positively correlated with *CCND1* levels in both ER-positive breast cancer cell lines and patient tumor tissues. Overall, our findings reveal the critical role of circFOXK2 in stabilizing the oncogene *CCND1* and promoting cancer progression, positioning circFOXK2 as a potential therapeutic target for ER-positive breast cancer in clinical settings.

## Significance

Estrogen receptor (ER)-positive breast cancer often develops resistance to endocrine therapies like tamoxifen. Understanding the molecular mechanisms underlying both carcinogenesis and drug resistance is critical for improving therapeutic strategies. This study identifies circFOXK2 as a key regulator of CCND1 in ER-positive breast cancer. By stabilizing *CCND1* mRNA through RNA–RNA pairing and recruitment of ELAVL1/HuR, circFOXK2 enhances the CCND1–CDK4/6–p-RB–E2F axis, promoting cell proliferation and therapy resistance. Genetic silencing and pharmacological inhibition of circFOXK2 suppress tumor growth and sensitize or restore tamoxifen responsiveness in ER-positive breast cancer. These findings highlight circFOXK2 as a potential therapeutic target for overcoming resistance and improving outcomes in ER-positive breast cancer patients, offering avenues for clinical intervention.

According to the latest global cancer statistics analysis, breast cancer is still the most commonly diagnosed cancer and the leading cause of cancer death in women (1). About 70% of breast cancer belongs to the ER-positive subtype (2–4), and endocrine therapy is the frontline of treatment for patients with ER-positive breast cancer at present. However, a significant proportion of patients face primary or acquired resistance (5–7).

The *CCND1* gene is a well-studied oncogene that encodes G1/S-specific cyclin CCND1. It is widely accepted that CCND1 forms a complex with CDK4/6, which phosphorylates tumor suppressor retinoblastoma (RB) to induce the release of transcription factors E2Fs in early G1 phase (2, 8–12). E2Fs further activate a large set of E2F-target genes to promote cells entering the S phase. Studies have shown that reduction of CCND1 expression is an early and critical event in endocrine treatment (13, 14). However, CCND1 overexpression is frequently observed in approximately 50% of breast cancers, with around 15% resulting from gene amplification (15–19), while others are driven by alternative signaling pathways such as phosphoinositide-3 kinase (PI3K) or through posttranscriptional regulation (2). Uncontrolled cell cycle progression caused by overexpression of CCND1 is one of the reasons for poor prognosis and resistance to endocrine therapy in ER-positive breast cancer (20–22). Thus, targeting CCND1 alone or in combination with endocrine therapy might be an effective strategy for treating ER-positive breast cancer.

Circular RNAs (circRNAs) are distinct from other RNA species in that the otherwise free 5′ and 3′ ends are covalently linked, resulting in a closed loop structure and making it exempt from endonuclease degradation (23–25). A growing number of studies have demonstrated that circRNAs are tightly associated with pathological processes, with implications in diagnosis and treatment of diseases (26–29). RNA–RNA interaction between circRNA and miRNA is the most common mode of action of circRNA (25, 28, 30, 31). Our previous research revealed that circPVT1 stabilizes *ESR1* mRNA to promote breast tumorigenesis and endocrine resistance in ER-positive breast cancer through acting as a competing endogenous RNA (ceRNA) to sponge miR-181a-2-3p (32). A recent study showed that circZNF609 binds to *CKAP5* mRNA directly to enhance *CKAP5* translation (33). However, whether circRNA regulates CCND1 mRNA, whether targeting it can suppress ER-positive breast cancer cell growth, and whether this approach can overcome endocrine therapy resistance remain unclear.

Here, we demonstrate that in ER-positive breast cancer cells, circFOXK2 is highly expressed and directly binds to *CCND1* mRNA via RNA–RNA pairing. This interaction recruits ELAVL1, which stabilizes *CCND1* mRNA, leading to increased CCND1 protein level and promoting uncontrolled G1/S cell cycle progression and cell growth. ASO specifically targeting circFOXK2 (ASO-circFOXK2) can effectively inhibit ER-positive breast tumor growth, and combination treatment with ASO-circFOXK2 and endocrine therapy drugs such as tamoxifen exhibits synergistic effects. Furthermore, ASO-circFOXK2 resensitizes tamoxifen-resistant ER-positive breast cancer cells to tamoxifen treatment.

## Results

### CircFOXK2 Promotes ER-Positive Breast Cancer Cell Growth Both In Vitro and In Vivo.

**Fig. 1.**
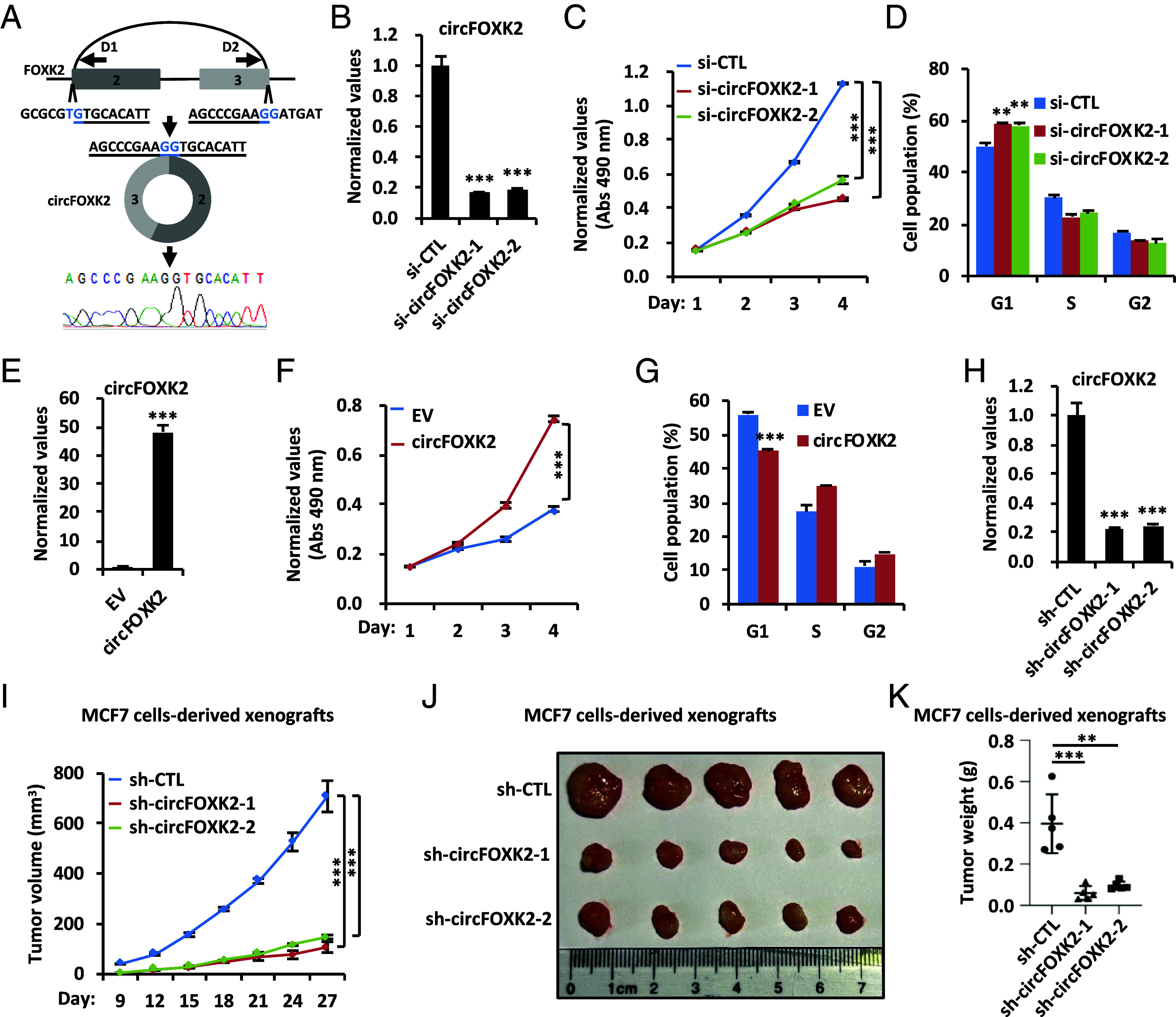
CircFOXK2 is required for ER-positive breast cancer cell growth both in vitro and in vivo. (*A*) Standard PCR was performed by using divergent primer sets flanking the junction region of circFOXK2, followed by Sanger sequencing. The sequences flanking the junction region are shown. The junction site is highlighted in light blue. The Sanger sequencing histogram is shown at the bottom. D1: divergent primer 1; D2: divergent primer 2. (*B*–*D*) MCF7 cells transfected with control siRNA (si-CTL) or two independent siRNAs specifically targeting circFOXK2 (si-circFOXK2-1 and si-circFOXK2-2) were subjected to RT-qPCR (*B*), cell proliferation (*C*), and FACS (*D*) analysis (±SD, **P < 0.01, ***P < 0.001). (*E*–*G*) MCF7 cells transfected with empty vector (EV) or vector expressing circFOXK2 were subjected to RT-qPCR (*E*), cell proliferation (*F*), and FACS (*G*) analysis (±SD, ***P < 0.001). (*H*) MCF7 cells infected with control shRNA (sh-CTL) or two independent shRNAs specifically targeting circFOXK2 (sh-circFOXK2-1 and sh-circFOXK2-2) were subjected to RT-qPCR (±SD, ***P < 0.001). (*I*–*K*) MCF7 cells infected with sh-CTL, sh-circFOXK2-1, or sh-circFOXK2-2 as described in (*H*) were injected subcutaneously into female BALB/C nude mice for xenograft assay. The growth curve (*I*), image (*J*), and weight (*K*) of tumors are shown (n = 5, ±SD, **P < 0.01, ***P < 0.001).

Recently, we reported the expression profile of circular RNAs (circRNAs) in the ER-positive breast cancer cell line MCF7 using circRNA sequencing (circRNA-seq) (32). CircFOXK2 (hsa_circ_0000816), one of the 50 most highly expressed circRNAs, drew our attention due to its parental gene, *FOXK2*, which exhibits diverse effects in various cancers (34, 35). CircFOXK2 with a length of 343 base pairs (bps) originates from the second and third exon of the *FOXK2* gene (Fig. 1*A*). Knockdown of circFOXK2 in MCF7 cells reduced cell growth and induced G1 phase cell cycle arrest (Fig. 1 *B*–*D*). We noticed that knockdown of circFOXK2 did not affect its parental gene FOXK2 expression at either the transcriptional or translational level (*SI Appendix*, Fig. S1 *A* and *B*). In contrast, overexpression of circFOXK2 promoted cell growth and G1/S phase progression in MCF7 cells (Fig. 1 *E*–*G*). The essential role of circFOXK2 in cell growth and cell cycle progression was independently confirmed in another ER-positive breast cancer cell line, T47D (*SI Appendix*, Fig. S1 *C*–*H*). CircFOXK2 also promoted cell growth in MCF10A cells, a normal human breast epithelial cell line (*SI Appendix*, Fig. S1 *I* and *J*). The oncogenic role of circFOXK2 was further demonstrated in MCF7 cell-derived xenograft mouse models, where circFOXK2 knockdown significantly reduced tumor size (Fig. 1 *H*–*K*) without affecting the body weight of the mice (*SI Appendix*, Fig. S1*K*). Again, knockdown of circFOXK2 did not affect its parental gene FOXK2 expression at either the transcriptional or translational level (*SI Appendix*, Fig. S1 *L* and *M*). Altogether, these data suggest that circFOXK2 promotes the growth of ER-positive breast cancer cells both in vitro and in vivo.

### CircFOXK2 Positively Regulates CCND1 Expression to Activate E2F Target Genes and Promote G1/S Transition in the Cell Cycle.

**Fig. 2.**
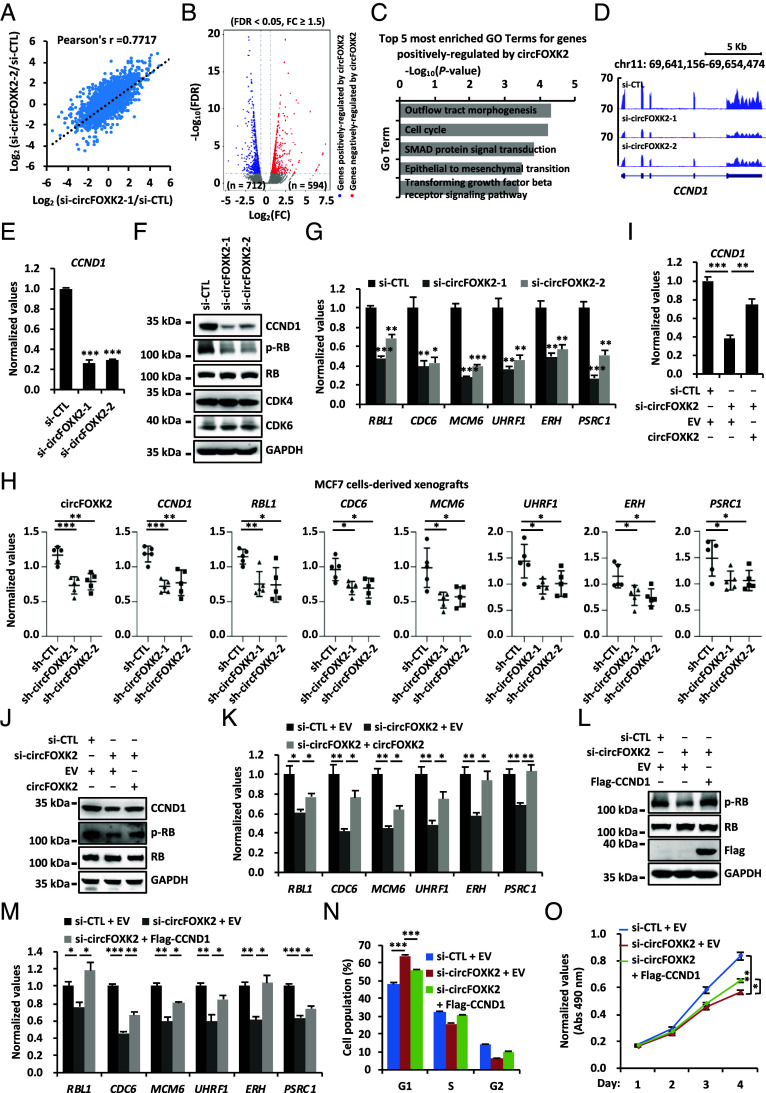
CircFOXK2 positively regulates the expression of CCND1 to activate E2F target genes and promote G1/S transition. (*A*) MCF7 cells were transfected with control siRNA (si-CTL) or two individual siRNAs specific against circFOXK2 (si-circFOXK2-1 and si-circFOXK2-2) followed by RNA-seq analysis. The correlation between the effects of the two si-circFOXK2 on the whole transcriptome is shown (Pearson correlation coefficient = 0.7717). (*B*) Genes that are positively and negatively regulated by circFOXK2 based on RNA-seq analysis as described in (*A*) are shown by volcano plot (FDR < 0.05, FC ≥ 1.5). (*C*) The five most enriched GO terms for genes positively regulated by circFOXK2 are shown. (*D*) UCSC genome browser view of RNA-seq as described in (*A*) for *CCND1* is shown. (*E*–*G*) MCF7 cells transfected with si-CTL, si-circFOXK2-1, or si-circFOXK2-2 were subjected to RT-qPCR (*E* and *G*) and immunoblotting (IB) (*F*) analysis (±SD, *P < 0.05, **P < 0.01, ***P < 0.001). Molecular weight is indicated (Kilodalton: kDa). (*H*) Tumor samples as described in Fig. 1*J* were subjected to RNA extraction and RT-qPCR analysis (n = 5, ±SD, *P < 0.05, **P < 0.01, ***P < 0.001). (*I*–*K*) MCF7 cells transfected with si-CTL or si-circFOXK2 in the presence of EV or vector expressing circFOXK2 were subjected to RT-qPCR (*I* and *K*) and IB (*J*) analysis (±SD, *P < 0.05, **P < 0.01, ***P < 0.001). (*L*–*O*) MCF7 cells transfected with si-CTL or si-circFOXK2 in the presence of EV or vector expressing Flag-tagged CCND1 were subjected to IB (*L*), RT-qPCR (*M*), FACS (*N*), and cell proliferation (*O*) analysis (±SD, *P < 0.05, **P < 0.01, ***P < 0.001).

To elucidate the molecular mechanism underlying circFOXK2 regulation of ER-positive breast cancer cell growth and tumorigenesis, we performed gene expression profiling to identify genes regulated by circFOXK2 in MCF7 cells. The results showed that the impact of both siRNAs targeting circFOXK2 on the whole transcriptome is well correlated (Pearson correlation coefficient = 0.7717) (Fig. 2*A*). There were 712 and 594 genes that were positively and negatively regulated by circFOXK2, respectively (Fig. 2*B* and *SI Appendix*, Table S1). Gene ontology (GO) analysis results indicated that “Cell cycle” is among the top five most enriched terms for genes positively regulated by circFOXK2 (Fig. 2*C* and *SI Appendix*, Table S1). Among all these cell cycle genes, *CCND1* was one of the most significantly regulated and closely related to G1/S progression in cell cycle (Fig. 2*D*). We first confirmed that the CCND1 expression decreased following circFOXK2 depletion at both the mRNA and protein level in both MCF7 (Fig. 2 *E* and *F*) and T47D cells (*SI Appendix*, Fig. S2 *A* and *B*). Phosphorylation of RB (p-RB) by CCND1–CDK4/6 complex is a critical step in releasing E2F from RB, leading to the activation of E2F target genes and the G1/S transition. Downregulation of CCND1 will lead to decreased levels of p-RB and subsequent downregulation of E2F-target genes. We observed that knockdown of circFOXK2 reduced the levels of p-RB (Fig. 2*F* and *SI Appendix*, Fig. S2*B*). Furthermore, we found that E2F target genes, such as *RBL1*, *CDC6*, *MCM6*, *UHRF1*, *ERH*, and *PSRC1*, were downregulated upon circFOXK2 knockdown in both MCF7 and T47D cells (Fig. 2*G* and *SI Appendix*, Fig. S2*C*). It should be noted that the expression of CDK4 and CDK6 was not affected by circFOXK2 knockdown (Fig. 2*F*). To support the functional importance of these circFOXK2-regulated, E2F target genes in tumor development, the expression of these genes was found to be significantly inhibited when circFOXK2 was knocked down in MCF7 cells-derived xenografts (Figs. 1*J* and 2*H*). As expected, reintroduction of circFOXK2 rescued the reduced expression of CCND1 at both mRNA and protein levels, the decreased p-RB levels, and the diminished expression of E2F target genes caused by circFOXK2 knockdown in both MCF7 and T47D cells (Fig. 2 *I*–*K* and *SI Appendix*, Fig. S2 *D*–*F*).

To support that circFOXK2 regulates G1/S transition and cell growth by targeting *CCND1*, introduction of CCND1 rescued the decreased levels of p-RB and the diminished expression of E2F target genes caused by circFOXK2 knockdown in MCF7 cells (Fig. 2 *L* and *M*). Furthermore, introduction of CCND1 rescued the cell cycle and growth defects caused by circFOXK2 knockdown (Fig. 2 *N* and *O*). Altogether, these data suggest that circFOXK2 regulates CCND1 expression to promote G1/S transition in the cell cycle.

### CircFOXK2 Pairs with the 3′ UTR of *CCND1* mRNA and Recruits ELAVL1 to Stabilize the *CCND1* mRNA.

**Fig. 3.**
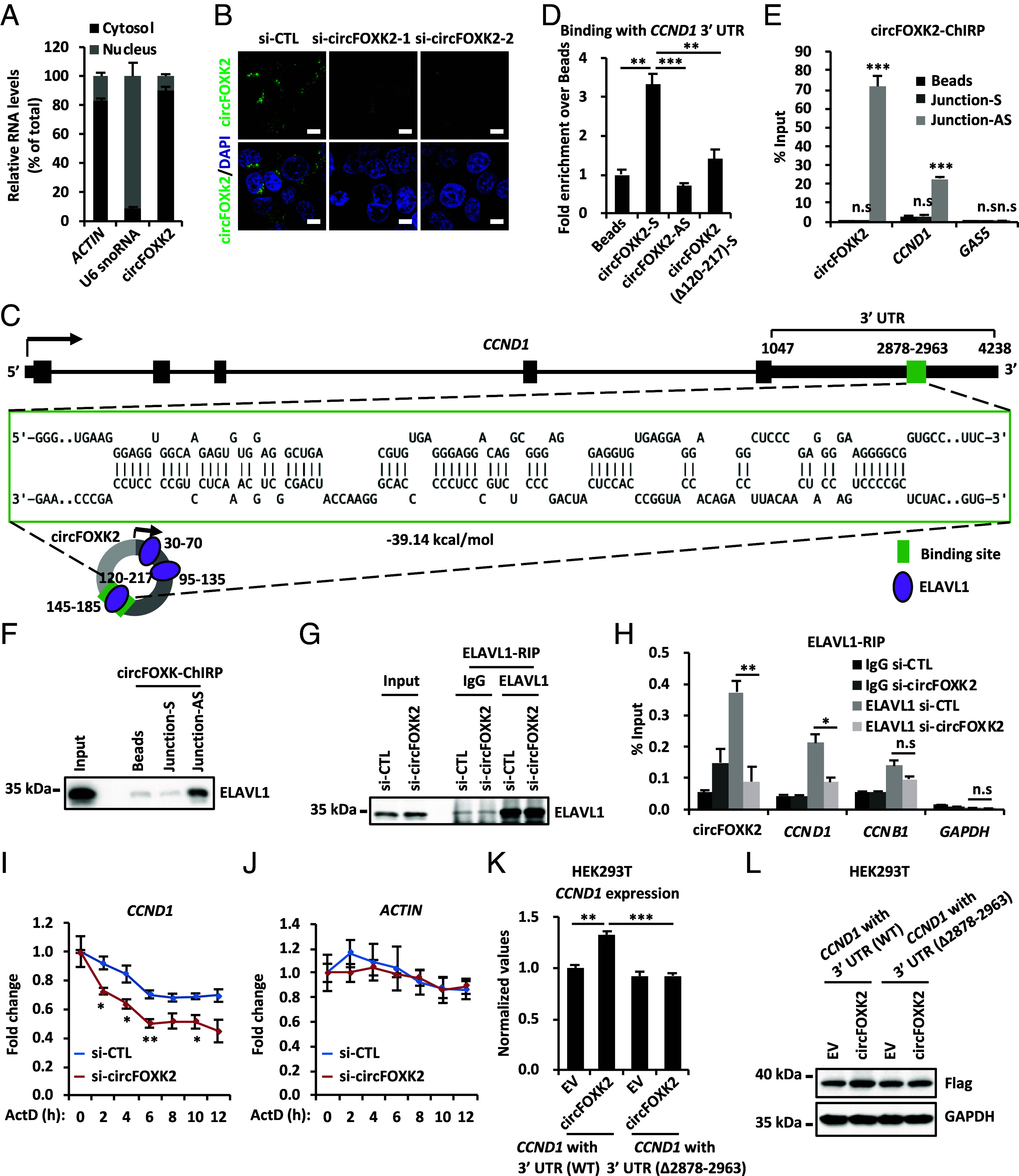
CircFOXK2 pairs with the 3′ UTR of *CCND1* mRNA and recruits ELAVL1 to stabilize *CCND1* mRNA. (*A*) MCF7 cells were subjected to cellular fractionation followed by RNA extraction and RT-qPCR analysis for genes as indicated. (*B*) MCF7 cells transfected with control siRNA (si-CTL) or two individual siRNAs specific against circFOXK2 (si-circFOXK2-1 and si-circFOXK2-2) were subjected to RNA-FISH analysis using probes specifically targeting circFOXK2. Nuclei was stained with DAPI. Green: circFOXK2; Blue: DAPI. (Scale bar, 5 μm.) (*C*) Schematic representation of the predicted RNA–RNA interaction regions between circFOXK2 and the 3′ UTR of *CCND1*. (*D*) In vitro RNA–RNA interaction assay was performed by incubating streptavidin C1 Dynabeads with or without the 3′ UTR of *CCND1* in the presence or absence of biotin-labeled, sense circFOXK2 (circFOXK2-S), antisense circFOXK2 (circFOXK2-AS), or sense circFOXK2 with the predicated *CCND1*-binding region deleted (circFOXK2 (∆120-217)-S). The precipitated RNAs were measured by RT-qPCR analysis using primers specifically targeting the 3′ UTR of *CCND1*. Data were normalized to the amount of RNA pulled down from empty beads (±SD, **P < 0.01, ***P < 0.001). (*E* and *F*) ChIRP assay was performed by incubating cell lysates prepared from MCF7 cells with or without sense (Junction-S) or antisense (Junction-AS) probe specifically targeting the junction region of circFOXK2 followed by RT-qPCR (*E*) and IB (*F*) analysis (±SD, n.s: nonsignificant, ***P < 0.001). (*G* and *H*) RIP assay was performed by incubating cell lysates prepared from MCF7 cells transfected with si-CTL or si-circFOXK2 with control IgG or anti-ELAVL1 antibody, followed by IB (*G*) and RT-qPCR (*H*) analysis (±SD, n.s: nonsignificant, *P < 0.05, **P < 0.01). (*I* and *J*) MCF7 cells were transfected with si-CTL or si-circFOXK2 and then treated with or without Actinomycin D (ActD, 10 μg/mL) for duration as indicated, followed by RT-qPCR analysis to examine the expression of *CCND1* (*I*) and *ACTIN* (*J*) (±SD, *P < 0.05, **P < 0.01). (*K* and *L*) HEK293T cells were transfected with EV or vector expressing circFOXK2 in the presence or absence of Flag-tagged, wild-type (WT) CCND1 or CCND1 with the circFOXK2-binding region in the 3′ UTR deleted (∆2878–2963) for 48 h followed by RT-qPCR (*K*) and IB (*L*) analysis (±SD, **P < 0.01, ***P < 0.001).

In order to further explore how circFOXK2 regulates the expression of *CCND1*, we first examined the localization of circFOXK2 through both cellular fractionation and FISH analysis. The results showed that it is mainly located in the cytoplasm (Fig. 3 *A* and *B*). One of the general mechanisms for cytosolic circRNAs to regulate mRNA abundance is to act as a competing endogenous RNA (ceRNA), such that they compete with mRNAs to bind with miRNAs. We therefore constructed a ceRNA network for circFOXK2, but without returning any miRNAs that are predicted to target both circFOXK2 and *CCND1*. An alternative way of noncoding RNAs to regulate mRNA biology is through direct RNA–RNA interaction (33, 36–41). By using IntraRNA website analysis (42–45), the 3′ UTR of *CCND1* (nucleotides 2878–2963) is predicted to be able to pair with circFOXK2 (nucleotides 120–217) (Fig. 3*C*). The direct interaction between circFOXK2 and the 3′ UTR of *CCND1* was confirmed by an RNA–RNA interaction assay in vitro (Fig. 3D). Depletion of nucleotides 120–217 in circFOXK2 significantly reduced the interaction between circFOXK2 and the 3′ UTR of *CCND1* (Fig. 3*D*). To further prove the interaction between circFOXK2 and *CCND1*, ChIRP-qPCR assay was performed in MCF7 cells by using an antisense probe specifically targeting the back-splicing junction of circFOXK2. The results showed that *CCND1* was pulled down, whereas *GAS5* exhibited no binding (Fig. 3*E*). To serve as a negative control, none of circFOXK2, *CCND1*, or *GAS5* was pulled down by the sense probe (Fig. 3*E*). Notably, the *CCND1* mRNA binding site of circFOXK2 is not located at the back-splicing junction but rather near conventional splicing sites between exon 2 and exon 3 of *FOKX2*. To assess whether linear *FOXK2* mRNA can bind to *CCND1* mRNA, the ChIRP-qPCR assay was performed in MCF7 cells using antisense probes specifically targeting *FOXK2*. The results showed that *FOXK2*, but not *CCND1* mRNA, was efficiently pulled down (*SI Appendix*, Fig. S3*A*). To further exclude the possibility that the defects in cell growth and G1/S phase progression upon circFOXK2 knockdown are due to FOXK2 interference, we found that FOXK2 knockdown led to enhanced *CCND1* expression at both the mRNA and protein levels, increased levels of p-RB, elevated expression of E2F target genes, and accelerated cell growth in MCF7 cells (*SI Appendix*, Fig. S3 *B*–*D*). These results suggest that circFOXK2 and FOXK2 play opposing roles in regulating CCND1.

Next, we aimed to explore how the direct interaction between circFOXK2 and *CCND1* regulates *CCND1* expression. It has been reported that the RNA binding protein (RBP) ELAVL1/HuR, a member in the ELAV-like RBP family, selectively binds to the AU-rich elements in the 3′ UTR of mRNAs and stabilizes mRNAs (33, 46, 47). CircInteractome database (48) indicated that circFOXK2 has three binding sites for ELAVL1 (Fig. 3*C*). In addition, ELAVL1 has been show to target *CCND1* mRNA through photoactivatable-ribonucleoside-enhanced cross-linking and immunoprecipitation (PAR-CLIP) (49). Thus, we first tested whether circFOXK2 binds to ELAVL1 through chromatin isolation by RNA purification-IB (ChIRP-IB) analysis. The results indicated that ELAVL1 was pulled down by the antisense probe targeting circFOXK2, but not the sense probe (Fig. 3*F*). RNA immunoprecipitation (RIP) assay with anti-ELAVL1 specific antibody showed that ELAVL1 could pull down both circFOXK2 and *CCND1* (Fig. 3 *G* and *H*). Interestingly, the enrichment of *CCND1* was significantly reduced upon circFOXK2 knockdown in MCF7 cells (Fig. 3*H*). However, the enrichment of *CCNB1*, a gene encoding a cyclin protein reported to bind with ELAVL1 (49, 50), was not altered by circFOXK2 knockdown (Fig. 3*H*). As a negative control, *GAPDH* mRNA showed no binding with ELAVL1 (Fig. 3*H*). Thus, we propose that circFOXK2 recruits ELAVL1 to stabilize *CCND1* mRNA. To test this, we treated control and circFOXK2-knockdown MCF7 cells with Actinomycin D (ActD) to inhibit transcription, followed by measurement of mRNA stability. The results showed that the stability of *CCND1* mRNA was significantly decreased upon circFOXK2 knockdown (Fig. 3*I*). To serve as a negative control, the stability of *ACTIN* mRNA was not affected (Fig. 3*J*). However, after knockdown of ELAVL1, circFOXK2 no longer regulates the stability of *CCND1* mRNA (*SI Appendix*, Fig. S3 *E*–*G*). This observation highlights a cooperative role between circFOXK2 and ELAVL1 in *CCND1* regulation. The copy number of circFOXK2 is comparable to that of *CCND1* and *FOXK2* in MCF7 cells (*SI Appendix*, Fig. S3 *H*–*K*). Similar as other circRNAs, circFOXK2 is much more stable than its parental gene *FOXK2* in the presence of RNase R and ActD (*SI Appendix*, Fig. S3 *L* and *M*). To further support that circFOXK2 can stabilize *CCND1* mRNA, the expression of Flag-tagged CCND1 with 3′ UTR (WT), but not *CCND1* (∆2878–2963), was significantly increased in the presence of circFOXK2 as measured at both RNA and protein levels (Fig. 3 *K* and *L*). Altogether, these data suggest that circFOXK2 directly binds to the 3′ UTR of *CCND1* mRNA and recruits ELAVL1 to stabilize the *CCND1* mRNA.

### Antisense Oligonucleotide (ASO) Targeting circFOXK2 Suppresses ER-Positive Breast Cancer Cell Growth Both In Vitro and In Vivo.

**Fig. 4.**
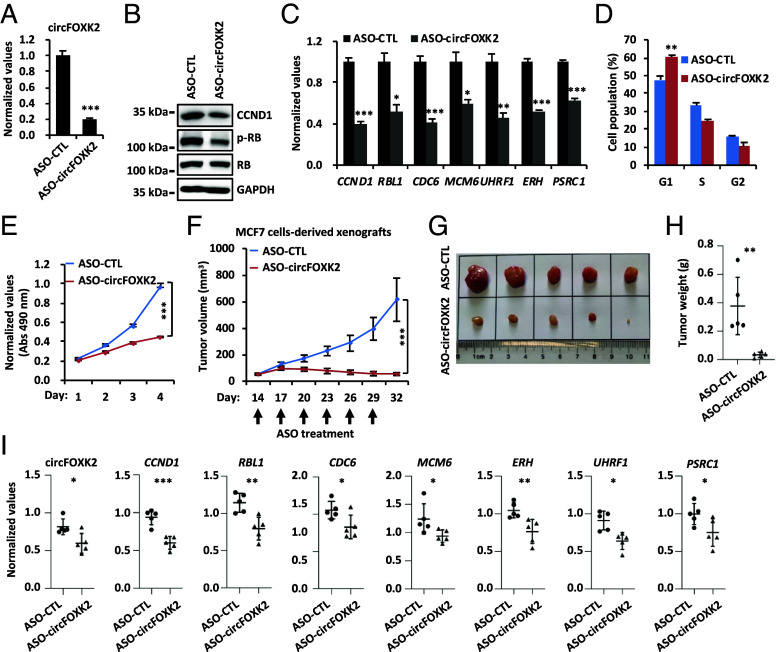
ASO targeting circFOXK2 suppresses ER-positive breast cancer cell growth both in vitro and in vivo. (*A*–*E*) MCF7 cells transfected with control ASO (ASO-CTL) or ASO specifically targeting circFOXK2 (ASO-circFOXK2) were subjected to RT-qPCR (*A* and *C*), IB (*B*), FACS (*D*), and cell proliferation (*E*) analysis (±SD, *P < 0.05, **P < 0.01, ***P < 0.001). (*F*–*H*) Mice inoculated subcutaneously with MCF7 cells were treated with ASO-CTL or ASO-circFOXK2 (5 nmol per dose, every 3 d) intratumorally for six times. The growth curve (*F*), image (*G*), and weight (*H*) of tumors are shown (n = 5, ±SD, **P < 0.01, ***P < 0.001). (*I*) Tumor samples as described in (*G*) were subjected to RNA extraction and RT-qPCR analysis (n = 5, ±SD, *P < 0.05, **P < 0.01, ***P < 0.001).

The functional importance of circFOXK2 promotes us to explore its potential as a therapeutic target for ER-positive breast cancer. ASOs can modulate gene expression, either by blocking translation or promoting the degradation of the RNA, which have been used for research and therapeutic purposes (51–53). We therefore designed an ASO specifically targeting circFOXK2 (ASO-circFOXK2), which effectively knocked down circFOXK2 in both MCF7 and T47D cells (Fig. 4*A* and *SI Appendix*, Fig. S4*A*). ASO-circFOXK2 did not affect the expression of FOXK2 (*SI Appendix*, Fig. S4 *B* and *C*). The expression of CCND1 at both the mRNA and protein levels, along with the levels of p-RB and the expression of E2F target genes, were significantly reduced in MCF7 and T47D cells treated with ASO-circFOXK2 (Fig. 4 *B* and *C* and *SI Appendix*, Fig. S4 *D* and *E*). Consequently, ASO-circFOXK2 treatment resulted in a delayed G1/S transition and a reduced cell proliferation rate (Fig. 4 *D* and *E* and *SI Appendix*, Fig. S4 *F* and *G*). Furthermore, ASO-circFOXK2 significantly inhibited tumor growth in MCF7 cell-derived xenografts (Fig. 4 *F*–*H*) without affecting the body weight of the mice (*SI Appendix*, Fig. S4*H*). Meanwhile, the expression of *CCND1* and E2F target genes was downregulated in ASO-circFOXK2-treated tumors (Fig. 4*I*), strengthening the functional importance of these genes in circFOXK2-mediated tumor growth. Altogether, these data suggest that ASO targeting circFOXK2 is effective in suppressing ER-positive breast cancer cells both in vitro and in vivo.

### Combination Treatment with ASO-circFOXK2 and Tamoxifen Exhibits Synergistic Effects in Suppressing ER-Positive Breast Cancer Cell Growth Both In Vitro and In Vivo.

**Fig. 5.**
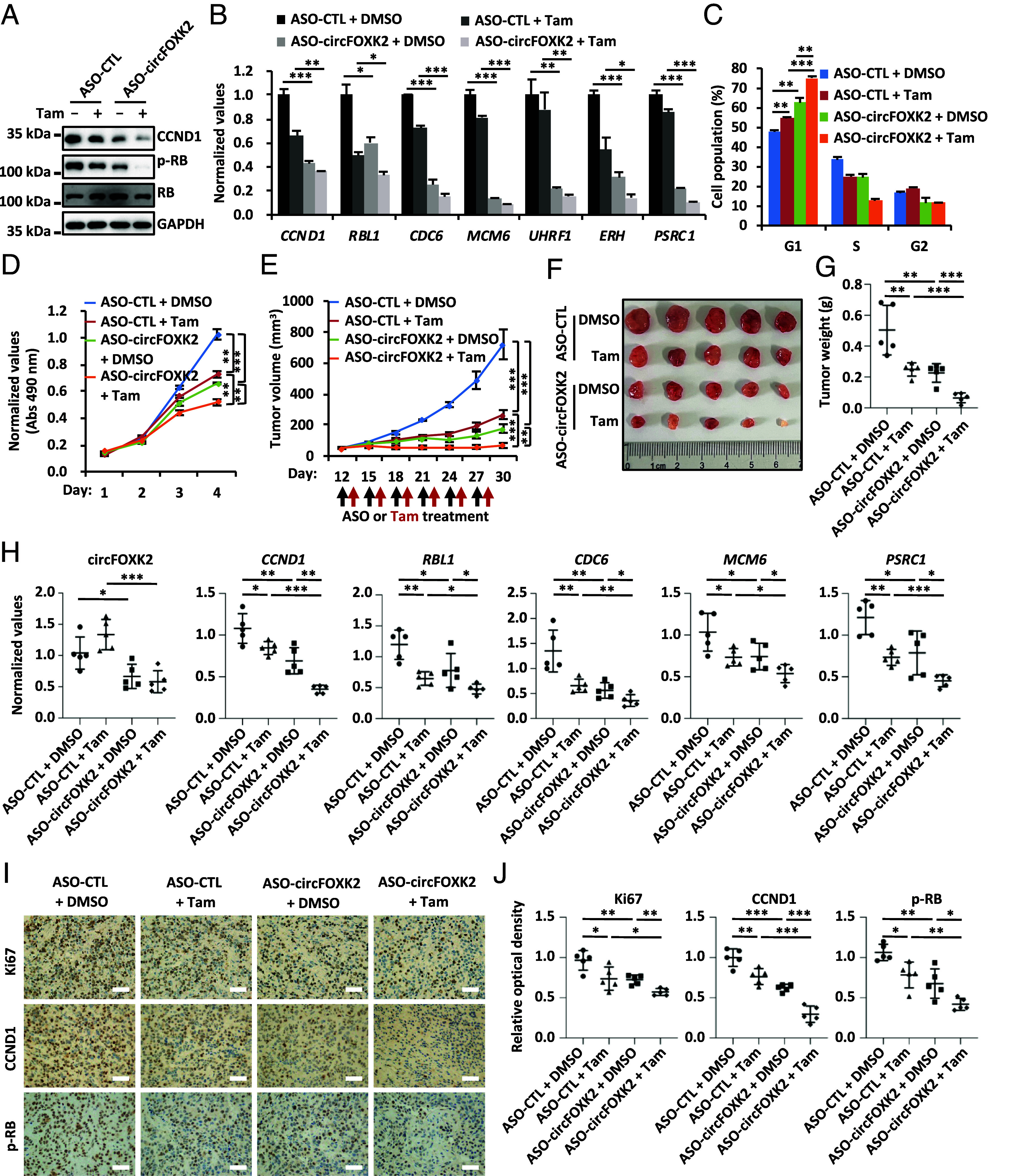
Combination treatment with ASO-circFOXK2 and tamoxifen shows synergistic effects in suppressing ER-positive breast cancer cell growth both in vitro and in vivo. (*A*–*C*) MCF7 cells were transfected with control ASO (ASO-CTL) or ASO specifically targeting circFOXK2 (ASO-circFOXK2) for 36 h before treating with or without tamoxifen (Tam, 5 μM) for 36 h, followed by IB (*A*), RT-qPCR (*B*), and FACS (*C*) analysis (±SD, *P < 0.05, **P < 0.01, ***P < 0.001). (*D*) MCF7 cells were transfected with ASO-CTL or ASO-circFOXK2 and then treated with or without tamoxifen (Tam, 5 μM), followed by cell proliferation assay (±SD, **P < 0.01, ***P < 0.001). (*E*–*G*) Female BALB/C nude mice were inoculated subcutaneously with MCF7 cells. Once the tumors were palpable, mice were treated intratumorally with either ASO-CTL or ASO-circFOXK2 (2.5 nmol per dose, every 3 d for six cycles). Tamoxifen (Tam, 20 mg/kg, every 3 d for six cycles) or vehicle were administrated intragastrically (i.g.) every other day. The growth curve (*E*), image (*F*), and weight (*G*) of tumors are shown (n = 5, ±SD, **P < 0.01, ***P < 0.001). (*H*) Tumor samples as described in (*F*) were subjected to RNA extraction and RT-qPCR analysis (n = 5, ±SD, *P < 0.05, **P < 0.01, ***P < 0.001). (*I*) Tumor samples as described in (*F*) were subjected to IHC analysis with antibodies against Ki67, CCND1, or p-RB, and representative images are shown (400× magnification). (Scale bar, 50 μm.) (*J*) The average optical density (OD) of the tumor areas as described in (*I*) was assessed using ImageJ, and results are presented as the relative staining intensity compared to control (n = 5, ±SD, *P < 0.05, **P < 0.01, ***P < 0.001).

Endocrine therapy is one of the most effective treatment options for ER-positive breast cancer. However, some patients remain resistant to this therapy. Amplification and overexpression of CCND1 is one of the main causes of primary endocrine therapy resistance in some ER-positive breast cancer patients (54, 55). As CCND1 itself as well as its downstream E2F target genes are major targets of tamoxifen, we therefore tested whether treatment with ASO-circFOXK2 will increase the sensitivity of tamoxifen in MCF7 cells. As expected, treatment with ASO-circFOXK2 and tamoxifen individually reduced the levels of CCND1, p-RB, and E2F target genes in both MCF7 and T47D cells (Fig. 5 *A* and *B* and *SI Appendix*, Fig. S5 *A* and *B*). Interestingly, cotreatment with ASO-circFOXK2 and tamoxifen exhibited synergistic effects on the levels of CCND1, p-RB, and E2F target genes (Fig. 5 *A* and *B* and *SI Appendix*, Fig. S5 *A* and *B*). Accordingly, cotreatment with ASO-circFOXK2 and tamoxifen exhibited synergistic effects on suppressing G1/S progression and cell growth compared to treatment with either ASO-circFOXK2 or tamoxifen individually in both MCF7 and T47D cells (Fig. 5 *C* and *D* and *SI Appendix*, Fig. S5 *C* and *D*). More importantly, ASO-circFOXK2 further enhanced the inhibitory effects of tamoxifen on tumor growth in MCF7 cells-derived xenograft mouse models (Fig. 5 *E*–*G*) without affecting the body weight of the mice (*SI Appendix*, Fig. S5*E*). As expected, the expression of *CCND1* and E2F target genes was the lowest in ASO-circFOXK2 and tamoxifen cotreated tumors (Fig. 5*H*). Immunohistochemistry (IHC) staining of tumor tissue sections further revealed that cotreatment with ASO-circFOXK2 and tamoxifen had synergistic effects in reducing the expression of Ki67 and CCND1, as well as the abundance of p-RB (Fig. 5 *I* and *J*). Altogether, these data suggest that combination treatment with ASO-circFOXK2 and tamoxifen had synergistic effects in suppressing ER-positive breast cancer cell growth both in vitro and in vivo, potentially leading to more effective treatment options for patients who are nonresponsive to standard endocrine therapies.

### ASO-circFOXK2 Resensitizes Tamoxifen-Resistant ER-Positive Breast Cancer Cells to Tamoxifen Treatment.

**Fig. 6.**
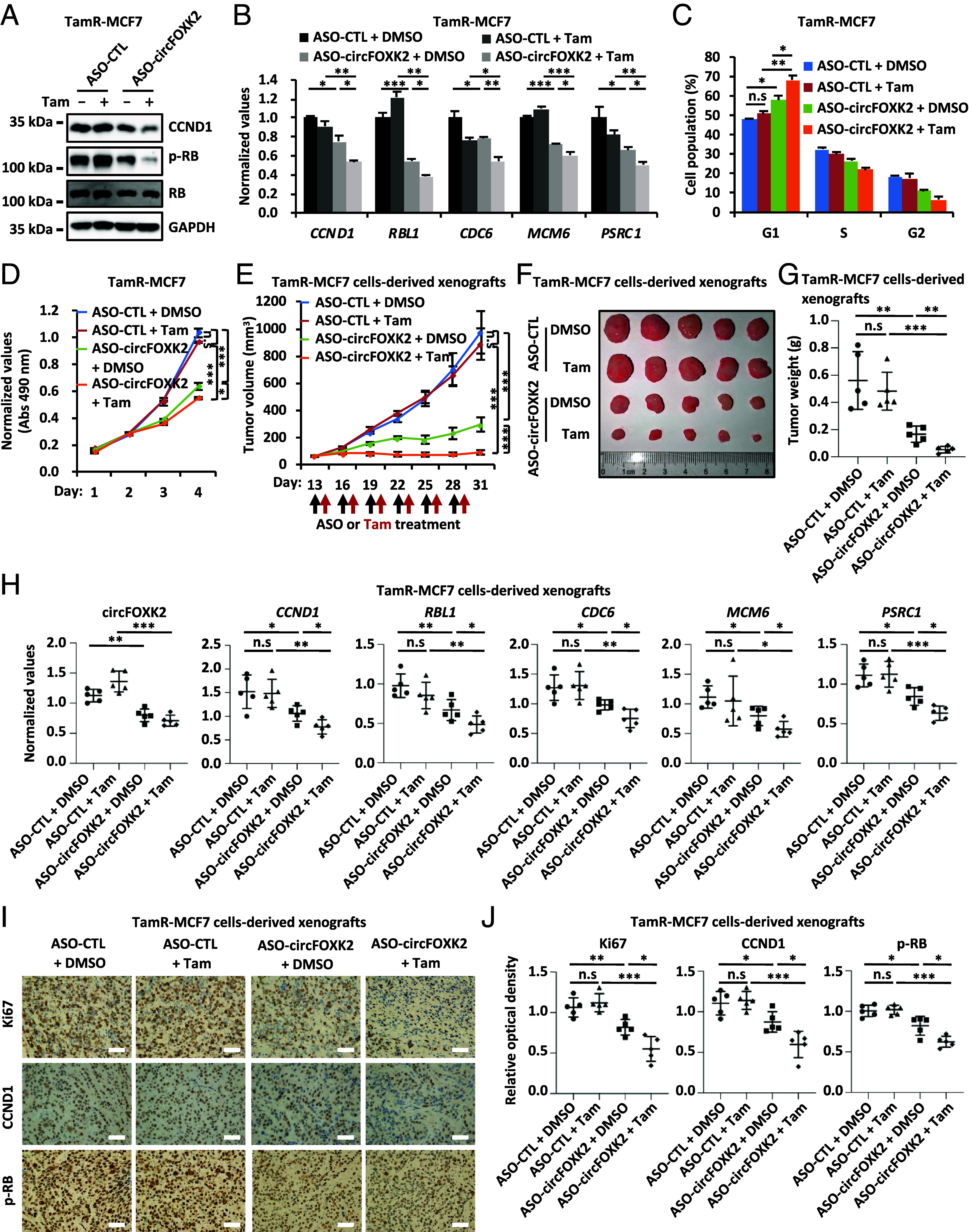
ASO-circFOXK2 resensitizes tamoxifen-resistant ER-positive breast cancer cells to tamoxifen treatment. (*A*–*C*) Tamoxifen-resistant MCF7 (TamR-MCF7) cells were transfected with control ASO (ASO-CTL) or ASO specifically targeting circFOXK2 (ASO-circFOXK2) for 36 h before treating with or without tamoxifen (Tam, 5 μM) for 36 h, followed by IB (*A*), RT-qPCR (*B*), and FACS (*C*) analysis (±SD, n.s: nonsignificant, *P < 0.05, **P < 0.01, ***P < 0.001). (*D*) TamR-MCF7 cells were transfected with ASO-CTL or ASO-circFOXK2 and treated with or without tamoxifen (Tam, 5 μM), followed by cell proliferation assay (±SD, n.s: nonsignificant, *P < 0.05, ***P < 0.001). (*E*–*G*) Female BALB/C nude mice were inoculated subcutaneously with TamR-MCF7 cells. Once the tumors were palpable, mice were treated intratumorally with either ASO-CTL or ASO-circFOXK2 (2.5 nmol per dose, every 3 d for six cycles). Tamoxifen (Tam, 20 mg/kg, every 3 d for six cycles) or vehicle were administrated intragastrically (i.g.) every other day. The growth curve (*E*), image (*F*), and weight (*G*) of tumors are shown (n = 5, ±SD, n.s: nonsignificant, **P < 0.01, ***P < 0.001). (*H*) Tumor samples as described in (*F*) were subjected to RNA extraction and RT-qPCR analysis (n = 5, ±SD, n.s: nonsignificant, *P < 0.05, **P < 0.01, ***P < 0.001). (*I*) Tumor samples as described in (*F*) were subjected to IHC analysis with antibodies against Ki67, CCND1, or p-RB, and representative images are shown (400× magnification). (Scale bar, 50 μm.) (*J*) The average OD of the tumor areas as described in (*I*) was assessed using ImageJ, and results are presented as the relative staining intensity compared to control (n = 5, ±SD, n.s: nonsignificant, *P < 0.05, **P < 0.01, ***P < 0.001).

Uncontrolled expression of CCND1 is often associated with endocrine therapy resistance in ER-positive breast cancer treatment. We therefore propose that targeting circFOXK2 might be effective in overcome endocrine therapy resistance in ER-positive breast cancer. We first confirmed that CCND1 expression was resistant to tamoxifen treatment in tamoxifen-resistant MCF7 cells (TamR-MCF7) (*SI Appendix*, Fig. S6 *A* and *B*), which was apparent different from that in parental MCF7 cells (Fig. 5 *A* and *B*). Furthermore, *CCND1* knockdown restored tamoxifen sensitivity in TamR-MCF7 cells, leading to a significant increase in G1 phase arrest and a much slower cell growth in the presence of tamoxifen (*SI Appendix*, Fig. S6 *C* and *D*). We then tested whether targeting circFOXK2 using ASO-circFOXK2 will restore tamoxifen sensitivity in TamR-MCF7 cells. As expected, tamoxifen had no effects on the levels of CCND1, p-RB, or circFOXK2-regulated E2F target genes in TamR-MCF7 cells (Fig. 6 *A* and *B*). Consequently, G1/S progression and cell growth were unaffected by tamoxifen treatment (Fig. 6 *C* and *D*). However, ASO-circFOKX2 treatment resensitized TamR-MCF7 cells to tamoxifen treatment, as evidenced by the reduced levels of CCND1, p-RB, and circFOXK2-regulated E2F target genes, along with impaired G1/S progression and cell growth (Fig. 6 *A*–*D*).

Furthermore, we explored whether targeting circFOXK2 will restore tamoxifen sensitivity in xenograft mouse models. TamR-MCF7 cells were inoculated subcutaneously into female BALB/C nude mice followed by ASO and/or tamoxifen treatment. In consistent with what we observed in cultured cells, ASO-circFOXK2 treatment restored TamR-MCF7 cells-derived xenografts to tamoxifen treatment, leading to a significant decrease of tumor growth (Fig. 6 *E*–*G*). The body weight of the mice was not affected (*SI Appendix*, Fig. S6*E*). The RT-qPCR and/or IHC analysis of tumor tissues revealed that ASO-circFOXK2 treatment restored the responses of CCND1 expression, RB phosphorylation, the expression of circFOXK2-regulated E2F target genes, and Ki67 expression to tamoxifen treatment (Fig. 6 *H*–*J*). Altogether, these data suggest that ASO targeting circFOXK2 is effective in overcoming tamoxifen resistance in ER-positive breast cancer cells, offering a potential strategy for improving clinical outcomes in patients facing acquired endocrine therapy resistance.

### CircFOXK2 Is Highly Expressed and Positively Correlated with *CCND1* in ER-Positive Breast Cancer and Other Cancer Types.

**Fig. 7.**
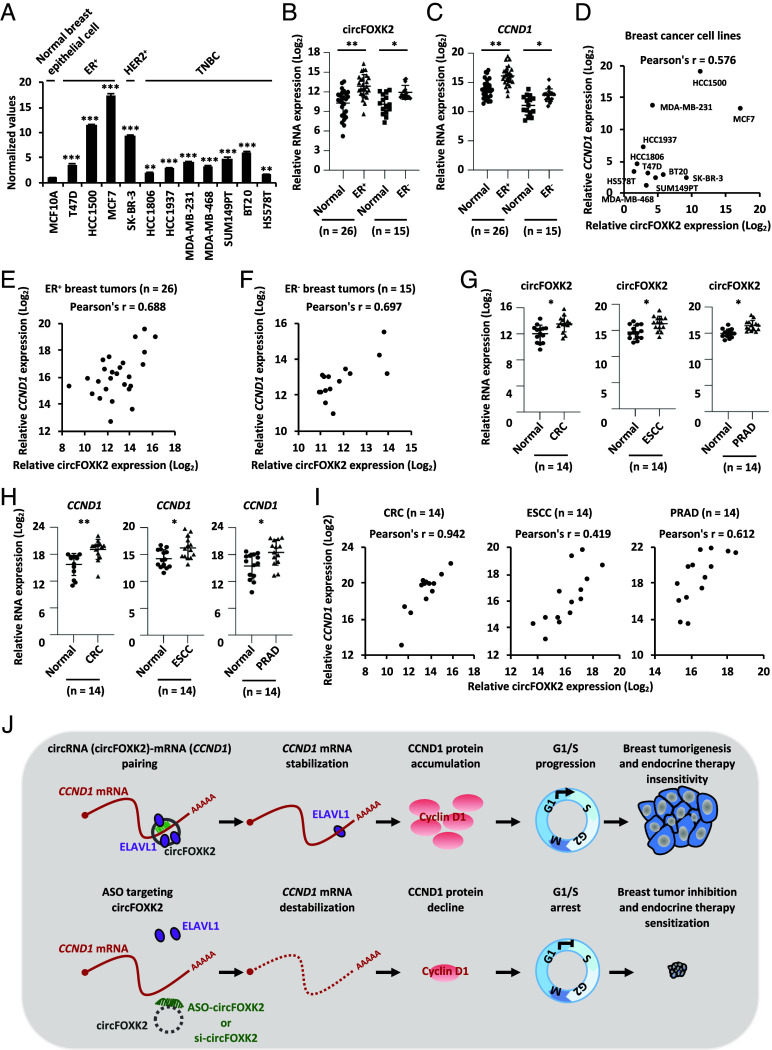
CircFOXK2 is highly expressed and positively correlated with *CCND1* in ER-positive breast cancer as well as other cancer types. (*A*) The expression of circFOXK2 was analyzed by RT-qPCR in a normal breast epithelial cell line and different subtypes of breast cancer cell lines as indicated (±SD, **P < 0.01, ***P < 0.001). (*B* and *C*) The expression of circFOXK2 (*B*) and *CCND1* (*C*) was analyzed by RT-qPCR in paired ER-positive (ER^+^, n = 26) or ER-negative (ER^−^, n = 15) breast tumor and adjacent normal breast tissues (±SD, *P < 0.05, **P < 0.01). (*D*–*F*) The correlation between the expression of circFOXK2 and *CCND1* in breast cancer cell lines (*D*) as well as ER^+^ (*E*) and ER^−^ (*F*) breast tumor tissues is shown. (*G* and *H*) The expression of circFOXK2 (*G*) and *CCND1* (*H*) was analyzed by RT-qPCR in paired CRC (n = 14), ESCC (n = 14), and PRAD (n = 14) tumor and adjacent normal tissues (±SD, *P < 0.05, **P < 0.01). (*I*) The correlation between the expression of circFOXK2 and CCND1 in CRC, ESCC, and PRAD tumor tissues is shown. (*J*) A proposed model depicts that elevated circFOXK2 in ER-positive breast cancer cells directly binds to the 3′ UTR of CCND1 mRNA and recruits ELAVL1 to stabilize CCND1 mRNA, leading to the accumulation of CCND1 protein, uncontrolled G1/S progression, and eventually breast tumorigenesis and tamoxifen resistance.

To investigate the clinical relevance of circFOXK2 in regulating *CCND1*, we first measured the expression of circFOXK2 in various breast cancer cell lines. We found that circFOXK2 was significantly higher in ER-positive breast cancer cell lines, including T47D, HCC1500, and MCF7, compared to normal breast epithelial cell line MCF10A (Fig. 7*A*). It should be noted that it is also highly expressed in HER2-positive and triple-negative breast cancer (TNBC) cell lines (Fig. 7*A*). Furthermore, circFOXK2 is significantly upregulated in both clinical ER-positive and ER-negative breast tumors compared to normal adjacent tissues (Fig. 7*B*). As expected, *CCND1* is also highly expressed in breast tumor samples (Fig. 7*C*). More importantly, circFOXK2 expression is positively correlated with that of *CCND1* in breast cancer cell lines and clinical breast tumor tissues (Fig. 7 *D*–*F*). Numerous studies have shown that CCND1 is overexpressed and/or amplified and thus oncogenic in a variety of tumors (56–63). Therefore, we examined the expression of circFOXK2 and *CCND1* as well as their association in other cancer types. The results showed that circFOXK2 and *CCND1* are expressed significantly higher in a number of cancer types we tested including colorectal cancer (CRC), esophageal squamous cell carcinoma (ESCC), and prostate cancer (PRAD) (Fig. 7 *G* and *H*). More importantly, the expression of circFOXK2 is highly correlated with that of *CCND1* (Fig. 7*I*). Altogether, these data suggest that circFOXK2 is highly expressed and positively correlated with *CCND1* in breast cancer and other cancer types.

## Discussion

Accumulated studies have demonstrated that overexpression of CCND1 is associated with aggressiveness and impaired tamoxifen responses in ER-positive breast cancers (55, 61). In this study, we identified circFOXK2 as a critical regulator of *CCND1* mRNA, which is highly expressed in ER-positive breast cancer and interacts with *CCND1* mRNA directly to increase its stability by recruiting the RNA-binding protein ELAVL1. Accumulated CCND1 leads to the aberrant activation of E2F target genes, G1/S progression, and cell growth. Accordingly, genetic silencing and pharmacological inhibition of circFOXK2 effectively suppressed tumor growth and enhance or restore tamoxifen sensitivity in ER-positive breast cancer (Fig. 7*J*).

A significant number of cytosolic circRNAs are found to function as competing endogenous RNAs (ceRNAs) to sponge-specific miRNAs and prevent miRNA-mediated degradation of mRNA targets (25, 28, 30, 31). However, the majority of circRNAs do not contain that many miRNA target sites so that they can function effectively as miRNA sponges (26, 64). It has been reported that circFOXK2 promotes pancreatic ductal adenocarcinoma (PDAC) progression by sponging miR-942 to stabilize the expression of *ANK1*, *GDNF*, and *PAX6* (65). Another report indicated that circFOXK2 interacts with miR-370 to promote breast cancer metastasis (66). A more recent study demonstrated that circFOXK2 works through the miR-149-3p/IL-6 axis to promote the tumorigenesis of non–small cell lung cancer (NSCLC) (67). Considering that circFOXK2 is primarily localized in the cytoplasm and plays an essential role in cell cycle progression, we analyzed its ceRNA network based on our RNA-seq analysis results. However, we failed to identify any circFOXK2-miRNA–mRNA hubs that are related to cell cycle regulation. RNA–RNA pairing including circRNA–mRNA has been shown to one of the many ways for circRNA to regulate gene expression (33, 40, 41). We therefore tested the possibility that circFOXK2 might directly bind to *CCND1* mRNA to regulate its expression. We performed a series of experiments to prove the binding between circFOXK2 and *CCND1* mRNA. Furthermore, three ELAVL1-binding sites were predicted on circFOXK2 and the binding among ELAVL1, cricFOXK2, and *CCND1* mRNA was confirmed experimentally. It should be noted that, in addition to cell cycle, GO terms such as epithelial to mesenchymal transition (EMT) and transforming growth factor beta (TGFβ) receptor signaling pathway are enriched in genes that are positively regulated by circFOXK2 based on our RNA-seq analysis. Therefore, whether and how circFOXK2 is involved in EMT and TGFβ signaling to promote cancer warrants future investigation.

Cancers often employ a variety of mechanisms to regulate CCND1 expression. One such mechanism is alternative polyadenylation (APA), which can lead to the shortening of the 3′ UTR (68, 69). This shortening produces *CCND1* isoforms with distinct 3′ UTR lengths, potentially altering the regulatory landscape. In some cases, shorter 3′ UTRs may result in the loss of circFOXK2 binding sites, thereby impairing circFOXK2-mediated stabilization of CCND1. Interestingly, a common G/A polymorphism (G/A870) in CCND1, which promotes the production of the cyclin D1b isoform, has been shown to possess oncogenic properties (70, 71). Compared to 3′ UTR shortening associated with APA, this polymorphism variant retains a circFOXK2 binding site. Therefore, circFOXK2 can still target and regulate the cyclin D1b isoform by binding to its 3′ UTR, leading to the reduction of cyclin D1b expression. This observation underscores the potential of circFOXK2 as a therapeutic target in cancer, highlighting its ability to modulate both full-length and some mutated CCND1 alterations, thus offering a potential avenue for treatment even in the presence of mutation-induced alterations.

CCND1 has been shown to be a critical downstream target gene of estrogen/ER-induced cell cycle progression and cell growth in ER-positive breast cancer (61, 72–74). Additionally, CCND1 is also well known as an oncogene in many other cancer types. Its amplification or overexpression has been implicated in CRC, PRAD, lung cancer, hepatocellular carcinoma (HCC), CRC, and ESCC, often driving uncontrolled cell proliferation, advanced disease stages, and poor patient prognosis. Furthermore, elevated CCND1 levels are frequently correlated with resistance to conventional therapies, further emphasizing the gene’s importance as a therapeutic target (56–63). Our study found that, beyond ER-positive breast cancer, circFOXK2 expression is up-regulated and strongly correlated with that of *CCND1* in ER-negative breast cancer, broadening the relevance of these findings beyond estrogen-driven pathways. Notably, circFOXK2 is also overexpressed in other cancers, including CRC, ESCC, and PRAD, with its expression again closely linked to that of *CCND1*. The molecular mechanisms underpinning CCND1 dysregulation in these cancers appear to be similar to those observed in breast cancer, suggesting that circFOXK2 may serve as a central modulator of CCND1 expression across various malignancies. Importantly, the conservation of these molecular mechanisms across cancer types offers an opportunity for the development of cross-cancer therapeutic strategies targeting circFOXK2 and CCND1. For example, in advanced PRAD in particular, CCND1 overexpression is associated with resistance to androgen deprivation therapy (ADT) (59, 62), a common therapeutic challenge, and targeting circFOXK2 and CCND1 could therefore be a promising strategy to overcome such resistance. Future studies investigating the precise molecular interactions between circFOXK2 and CCND1, as well as the potential for targeting this axis in clinical settings, could provide avenues for improving treatment outcomes across multiple cancer types.

Endocrine therapy is now the standard treatment for advanced hormone receptor (HR)-positive breast cancer (75). However, resistance to endocrine therapy is inevitable, which is largely due to the upregulation of CCND1 by hyperactivation of EGFR (76), PI3K (77, 78), and mTOR (79) pathways. Thus, combination therapy targeting these pathways has been shown to be effective in increasing the sensitivity of ER-positive breast cancer cells to endocrine therapy. Due to the intrinsic regulation between circFOXK2 and *CCND1*, we demonstrated that circFOXK2 plays a pivotal role in breast cancer progression and endocrine therapy resistance by stabilizing *CCND1* mRNA, thereby promoting CCND1 expression and facilitating tumor growth. This positions circFOXK2 as a potential therapeutic target, offering avenues to overcome endocrine therapy resistance. Specifically, the development of ASO targeting circFOXK2 represents a promising approach for clinical translation. Preclinical testing demonstrated that these ASOs effectively reduced breast cancer cell growth and reversed endocrine therapy resistance in vitro and in vivo. In addition to ASOs, circFOXK2 can be targeted using small-interfering RNA (siRNA), leveraging RNA interference (RNAi) mechanisms to silence gene expression. SiRNA therapeutics have emerged as an innovative drug class with significant potential in oncology and other therapeutic areas (80–82). Further optimization of delivery systems, such as lipid nanoparticles or exosome-based carriers, could enhance the clinical applicability of these approaches. Beyond its therapeutic potential, circFOXK2 expression could serve as a biomarker to identify patients who are most likely to benefit from circFOXK2-based therapies, particularly those with high circFOXK2 and CCND1 expression and resistance to endocrine treatments in breast cancer. On the other hand, unlike linear RNA, due to unique circular structure, circRNAs are stable and less prone to degradation in tissue samples and even in blood, making them ideal candidates for diagnostic markers. The expression of circFOXK2 is highly expressed in various cancers, and evaluating its expression in serum or plasma may provide a sensitive and specific way for cancer diagnosis. CircFOXK2 represents a promising diagnostic biomarker for breast cancer due to its high expression, stability, and correlation with disease progression. As research progresses, circFOXK2 could offer a noninvasive, reliable method for early diagnosis, prognosis prediction, and monitoring treatment responses in breast cancer patients. However, further validation and optimization of detection techniques are essential to fully realize its potential in clinical applications.

In summary, our study demonstrates that circFOXK2 function as a critical regulator of CCND1 expression through an RNA–RNA pairing mechanism, in which highly expressed circFOXK2 directly binds to *CCND1* mRNA and recruits ELAVL1 to stabilize *CCND1* mRNA, leading to the accumulation of CCND1 protein and thus aberrant expression of E2F target genes, G1/S progression, and cell growth as well as tamoxifen resistance in ER-positive breast cancer. Thus, circFOXK2 can serve as a potential diagnosis biomarker and therapeutic target for ER-positive breast cancer in the clinic.

## Materials and Methods

Detailed materials and methods are available in *SI Appendix*. This includes the following: clinical specimens, cell culture, RNA isolation and RT-qPCR, siRNA and ASO transfection, cloning procedures, lentivirus packaging and infection, cell proliferation assay, fluorescence-activated cell sorting (FACS) analysis, RNA-seq analysis, IB analysis, copy number analysis, RNase R digestion, RNA stability assay, cellular fractionation, RNA fluorescence in situ hybridization (RNA-FISH), in vitro RNA transcription and circularization, in vitro RNA–RNA interaction assay, ChIRP assay, RIP, xenograft tumor assay, IHC, and statistics analysis.

## Supplementary Material

Appendix 01 (PDF)

Dataset S01 (XLSX)

Dataset S02 (XLSX)

## Data Availability

RNA-seq data were deposited in the GEO database under Accession No. GSE284608 (83). All other data are included in the manuscript and/or supporting information.
