# Supplementary material for: A circRNA–mRNA pairing mechanism regulates tumor growth and endocrine therapy resistance in ER-positive breast cancer
Source: Proc Natl Acad Sci U S A. 2025 Feb 18;122(8):e2420383122. doi: 10.1073/pnas.2420383122 (PMC11874584; doi:10.1073/pnas.2420383122)
Supplement: Supplementary file 1 — Appendix 01 (PDF) [file pnas.2420383122.sapp.pdf]

Supporting Information for

A circRNA-mRNA pairing mechanism regulates tumor growth and endocrine therapy resistance in ER-positive breast cancer

Materials and methods

Clinical specimens

Breast tumor tissues and matched adjacent normal tissues were obtained from patients with breast cancer who accepted surgery at the Second Affiliated Hospital of Shantou University Medical College, which was approved by the Institutional Review Board of the Second Affiliated Hospital of Shantou University Medical College (ID: 2022-7). Colorectal cancer (CRC) and matched adjacent normal tissues were collected from patients with colorectal cancer who accepted surgery at the First Affiliated Hospital of Xiamen University, which was approved by the Institutional Review Board of the First Affiliated Hospital of Xiamen University (ID: XMYY-2023KYSB084). Esophageal squamous cell carcinoma (ESCC) tissues and matched adjacent normal tissues were obtained from patients with ESCC who accepted surgery at the Department of Thoracic Surgery of Fujian Medical University Union Hospital, which was approved by the Institutional Review Board of Fujian Medical University Union Hospital (ID: 2022-61). Prostate cancer (PRAD) tissues and matched adjacent normal tissues were obtained from patients with PRAD who accepted surgery at Ren Ji Hospital of Shanghai Jiao Tong University, which was approved by the Institutional Review Board of the Ren Ji Hospital, School of Medicine, Shanghai Jiao Tong University (ID: KY2022-200-

B). Tissue samples were freshly frozen in dry ice and stored at -80°C until RNA extraction. All research was performed in compliance with government policies and the Helsinki Declaration, and was approved by institutional review board at Xiamen University. Experiments were undertaken with the understanding and written consent of each subject.

Cell culture

Human breast cancer cell lines and embryonic kidney cell line HEK293T were obtained from ATCC and cultured in DMEM (Biological Industries) supplemented with 10% fetal bovine serum (Biological Industries) and 1% penicillin/streptomycin (Biological Industries). MCF10A cells were obtained from ATCC and cultured in MCF10A culture media (Procell) supplemented with 10% fetal bovine serum and 1% penicillin/streptomycin. Tamoxifen-resistant MCF7 (TamR-MCF7) were culture in DMEM with 10% fetal bovine serum, 1% penicillin/streptomycin, and 5 μM tamoxifen (MCE). Cells were maintained in a 5% CO_2 humidified incubator at 37°C.

RNA isolation and RT-qPCR

Total RNA was extracted from cells or tissues using TRIzol (Invitrogen) according to the manufacturer's instructions, and cDNA synthesis from total RNA was carried out using GoScript™ Reverse Transcription Mix with random primers (Promega). Quantitative real-time PCR (RT-qPCR) was performed using Hieff® qPCR SYBR Green Master Mix (Yeasen) and AriaMx Real-Time PCR machine (Agilent Technologies). All RT-qPCRs were repeated at least three times, and the relative abundance of each transcript was normalized to the expression level of *ACTIN*. All of

the primers used in the RT-qPCR assay are listed in Supplementary table 2.

Small-interfering RNA (siRNA) and anti-sense oligonucleotide (ASO) transfection

SiRNA and ASO (RiboBio) were transfected using Lipofectamine 2000 (Invitrogen) reagent according to the manufacturer's instructions. SiRNA-targeting sequence: GCCCGAAGGUGCACAUUCA (si-circFOXK2-1); CCCGAAGGUGCACAUUCAG (si-circFOXK2-2); GCAAGAACGGGGUAUUCGU (si-FOXK2); GUUGAAUCUGCAAAACUUA (si-ELAVL1); CCCGCACGAUUUCAUUGAA (si-CCND1); ASO-targeting sequence: ACAGCCCGAAGGUGCACAUU (ASO-circFOXK2). ASO was design as described previously[1].

Cloning procedures

Short hairpin RNA (shRNA)-targeting circFOXK2 was cloned into lenti-viral pLKO.1 vector with AgeI and EcoRI restriction enzymes. ShRNA-targeting sequence: CAGCCCGAAGGUGCACAUUCA (sh-circFOXK2-1); GCCCGAAGGUGCACAUUCAGG (sh-circFOXK2-2).

Full-length circFOXK2 was cloned in pCD2.1-ciR vector (Geneseed Biotech) with KpnI and BamHI restriction enzymes for overexpression. The complementary Alu sequences besides the restriction enzyme sites were responsible for back splicing. pCD2.1-circFOXK2 plasmid was transfected into cells by using LipoPlus DNA transfection reagent (Sagecreation). For circFOXK2 rescue assay, the back-splicing junction sequence "GCCCGAAGGTGCACATTCAGGTTC" was mutated to "GCCCGAAGGACCTCTTACAGGTTC" by overlap extension PCR method using

PrimeSTAR HS DNA Polymerase (Takara) to minimize siRNA-mediated degradation of exogenous circFOXK2.

Lenti-virus packaging and infection

Lenti-virus packaging and infection were performed as described previously[2]. Briefly, HEK293T cells were transfected with lenti-viral vectors together with packaging vectors, pMDL, VSVG, and REV, at a ratio of 10:5:3:2 using Polyethyleneimine (PEI; Polysciences) according to the manufacturer's instructions. Viral supernatant was harvested after 72 h by passing through a 0.45 μm filter. Virus was added to MCF7 cells in the presence of 10 μg/mL polybrene (Sigma-Aldrich), followed by centrifugation for 30 min at 1,500 g at 37°C.

Cell proliferation assay

Cell viability was measured by using a CellTiter 96 AQueous one solution cell proliferation assay kit (Promega) following the manufacturer's instructions. Briefly, cells were treated as indicated and maintained in culture medium for different time points followed by cell proliferation assay. Data was recorded at wavelength 490 nm using a Thermo Multiskan MK3 Microplate Reader.

Fluorescence-activated cell sorting (FACS) analysis

The cells were fixed in 75% cold ethanol for at least 24 h at -20°C and stained with 300-500 μL PI/Triton X-100 staining solution (0.1 % (v/v) Triton X-100, 0.2 mg/mL DNase-free RNase A, and 0.2 μg/mL PI). Cell cycle distribution was performed on a CytoFLEX LX flow cytometer (Beckman Coulter). The gating strategy in FlowJo was used to exclude cell debris and aggregates.

RNA-seq analysis

Total RNA was isolated using RNeasy Mini kit (Qiagen) following the manufacturer's instructions. DNase I in column digestion was included to ensure RNA quality. RNA library preparation was performed by using NEBNext® Ultra™ Directional RNA Library Prep Kit for Illumina (E7420L) and paired-end sequencing was performed with Illumina HiSeq 4000 platform. Low-quality reads were cleaned through Fastqc (version 0.11.9) and Fastp (version 0.22.0), two packages for sequence pre-disposal. Processed reads were aligned to human reference genome hg38 using STAR (version 2.7.11b). The alignment results were processed using the SourceForge Subread package featureCount (version 2.0.6) for gene quantification with the option -primary (count primary alignments only) -B (only count read pairs that have both ends aligned) -C (do not count read pairs that have their two ends mapping to different chromosomes or mapping to same chromosomes but on different strands) -Q 20 (the minimum mapping quality score 20) -s 0 (unstranded). Differential gene analysis was performed use edgeR (version 3.42.4), using FDR < 0.05 and FC ≥ 1.5 as cutoff. Then, volcano plots were drawn using ggplot2 (version 3.5.1) packages of R software to depict these differentially expressed genes. Go term analysis was performed using DAVID Bioinformatics website[3,4]. RNA-seq data were deposited in the Gene Expression Omnibus database under accession GSE284608.

Immunoblotting (IB) analysis

Cells were washed with ice-cold PBS twice and lysed in lysis buffer (50 mM Tris-HCl (pH 7.4), 150 mM NaCl, 1 mM EDTA, and 1% Triton X-100) supplemented with

protease inhibitor and phosphatase inhibitor on ice for 30 min. Lysates were centrifuged at 20,000 g for 10 min at 4°C. Protein concentration was measured by Bio-Rad protein assay (Bio-Rad). Equal amount of protein samples was boiled in SDS sample buffer and then subjected to SDS-PAGE and transferred to a nitrocellulose membrane (Millipore). Western blotting was performed following standard protocols. Antibodies used are listed as following: Rabbit polyclonal anti-FOXK2 (ABclonal, A14245, 1:2000); Rabbit polyclonal anti-CCND1 (Proteintech, 26939-1-AP, 1:2000); Rabbit monoclonal anti-p-RB (Ser780) (CST, D59B7, 1:2000); Rabbit monoclonal anti-p-RB (Ser780) (ABclonal, AP1419, 1:2000); Mouse monoclonal anti-RB (Santa Cruz Biotechnology, sc-73598, 1:2000); Rabbit polyclonal anti-CDK4 (Proteintech, 11026-1-AP, 1:2000); Rabbit polyclonal anti-CDK6 (Proteintech, 14052-1-AP, 1:2000); Rabbit monoclonal anti-ELAVL1 (ABclonal, A19622, 1:2000); Mouse monoclonal anti-GAPDH (Santa Cruz Biotechnology, sc-47724, 1:5000); Mouse monoclonal anti-Flag M2 (Sigma, F1804, 1:5000).

Copy number analysis

Copy number analysis was performed as described previously with minor modifications[5]. Briefly, reference standard was generated incorporating the cDNA sequence of circFOXK2, *CCND1*, or *FOXK2* in a corresponding PCR product. Absolute quantification was performed using 10-fold serial dilutions of the reference standard. Ct versus the dilution factor was plotted in a base-10 semi-logarithmic graph, fitting the data to a straight line. Plot was then used as a standard curve for extrapolating the number of molecules of circFOXK2, *CCND1*, or *FOXK2* in the cells.

RNase R digestion

Total RNAs were treated with RNase R (10 units/μg RNA, Epicentre) in RNase R buffer at 37°C for duration as indicated, followed by RT-qPCR analysis.

RNA stability assay

Cells were treated with 10 μg/mL Actinomycin D (MCE) for duration as indicated. RNA was extracted and followed by RT-qPCR analysis.

Cellular fractionation

Cellular fractionation was performed as described previously[6]. Briefly, cells were washed with ice-cold PBS, collected, spun down, and re-suspended in ice-cold buffer I (10 mM Hepes (pH 8.0), 1.5 mM $MgCl_2$, 10 mM KCl, and 1 mM DTT) supplemented with protease inhibitor cocktail and RNase inhibitor, followed by incubation for 15 min on ice. Igepal-CA630 was then added at a final concentration of 1% followed by vortexing for 10 sec. Nuclei were collected by centrifuging 3 min at maximum speed. The resultant supernatant was cytosolic fraction. Nuclei were then lysed in ice-cold buffer II (20 mM Hepes (pH 8.0), 1.5 mM $MgCl_2$, 25% glycerol, 420 mM NaCl, 0.2 mM EDTA, and 1 mM DTT) supplemented with protease inhibitor cocktail and RNase inhibitor, followed by centrifugation 15 min at maximum speed. The resultant supernatant was nuclear fraction. Both cytosolic and nuclear RNAs extracted with Phenol-Chloroform-Isoamyl Alcohol mixture (Sigma) was recovered by adding 1 μL of GlycoBlue (15 mg/mL, Ambion), 36 μL of 3 M sodium acetate, and 750 μL of ethanol, followed by incubation at -20°C for overnight. Precipitated RNAs were washed with 70% ethanol, air dried, and re-suspended in RNase free water, followed

by DNase I (Promega) treatment to remove genomic DNA. The resultant RNAs were subjected to RT-qPCR analysis.

RNA fluorescence in situ hybridization (RNA-FISH)

RNA-FISH assay was performed as described previously[2]. Briefly, cells grown on cover glass were fixed with fixation buffer (4% formaldehyde, 10% acetic acid) for 10 min, and permeabilized in 70% of ethanol overnight. Cells were then rehydrated in 2 × SSC buffer (300 mM NaCl and 30 mM sodium citrate (pH7.0)) with 50% formamide for 5 min. Hybridization solution, containing 30 ng Fam-labeled DNA probe (Fam-CCTGAATGTGCACCTTCGGGCTGTCTCCAC; Sangon Biotech), were added to the sample and incubated overnight at 37°C. The cells were washed twice for 30 min at 2 × SSC and 50% formamide solution. Nuclei were counterstained with DAPI (0.1 μg/mL). Three images of each cover glass were taken with Carl Zeiss laser confocal microscope, and representative images were shown.

***In vitro* RNA transcription and circularization**

In vitro RNA transcription was performed by T7 RNA Polymerase (Roche) to generate mRNA from double-stranded DNA templates carrying the T7 promotor sequence system following the manufacturer's instructions. Briefly, PCR-amplified T7-DNA fragments (200 ng) were incubated with T7 RNA polymerase enzyme (2 μL) and dNTP mix or Biotin RNA labeling mix (1 mM) for 2 h at 37°C, followed by DNase I treatment for 30 min at 37°C to remove DNA templates. Transcribed RNAs were precipitated with ethanol and re-suspended in RNase-free water.

For RNA circularization, *in vitro* transcribed linear RNAs (2 μg) were incubated with

T4 RNA ligase (Thermo Fisher) for 4 h at 37°C according to the manufacturer's protocol, followed by RNase R treatment for 2 h at 37°C to remove linear RNAs. Circularized RNAs were precipitated with ethanol and re-suspended in RNase-free water, and detected by RT-qPCR.

***In vitro* RNA-RNA interaction assay**

In vitro RNA-RNA interaction assay was performed as described previously with minor modifications[7]. Briefly, 5 pmol of biotin-labeled sense, anti-sense circFOXK2, or sense circFOXK2 with the predicated *CCND1*-binding region deleted (circFOXK2 (Δ120-217)-S) was incubated with or without the 3′ UTR of *CCND1* RNA fragment (2.5 pmol) for 4 h at 4°C. Following incubation, streptavidin C1 Dynabeads (5 μL, Invitrogen) were added into individual tubes and incubated 2 h at 4 °C. After incubation, beads were washed six times with RIP Buffer (100 mM KCl, 5 mM $MgCl_2$, 10 mM HEPES (pH 7.0), 0.5% NP-40, 1 mM DTT, and 2 mM vanadyl ribonucleoside complexes solution (Sigma)) supplemented with protease inhibitor cocktail and RNase inhibitor and added to the reaction. To recover RNA, beads were digested with proteinase K buffer supplemented with 1% SDS and 1.2 μg/μl proteinase K (Ambion) in RIP buffer at 55°C for 30 min with rotation. RNA was isolated using TRIzol, followed by RT-qPCR analysis.

Chromatin isolation by RNA purification (ChIRP) assay

Chromatin Isolation by RNA Purification (ChIRP) assay was performed as described previously with minor modifications[8]. Briefly, cells were fixed in 1% formaldehyde and lysed in lysis buffer (50 mM Tris-HCl (pH 7.0), 10 mM EDTA, and 1% SDS)

supplemented with 1 mM PMSF, protease inhibitor cocktail, and RNase inhibitor. The supernatant was collected by centrifugation at 16,100 g for 10 min at 4°C, and one volume of supernatant was diluted with two volume of hybridization buffer (750 mM NaCl, 1% SDS, 50 mM Tris-HCl (pH 7.0), 1 mM EDTA, and 15% formamide) supplemented with 1 mM PMSF, protease inhibitor cocktail, and RNase inhibitor, which were then incubated with 100 pM of biotin-labeled DNA probe targeting to the junction region of circFOXK2 (anti-sense: biotin-TCGGGAACCTGAATGTGCACCTTCGGGCTGTCTCCACCTG; sense: biotin-CAGGTGGAGACAGCCCGAAGGTGCACATTCAGGTTCCCGA) or *FOXK2* (anti-sense: biotin-TTGCCAACATGTGCAAAGGAC and biotin-CACCACAGAGTTGATATCGT; sense: biotin-GTCCTTTGCACATGTTGGCAA and biotin-ACGATATCAACTCTGTGGTG) at 37°C for 4 h with rotation. Then, streptavidin C1 Dynabeads (100 μL per 100 pmol of probes) was added to each sample and incubated at 37°C for 30 min with rotation. After incubation, beads were washed with lysis buffer for five times. Beads were then re-suspended in RNA PK buffer (100 mM NaCl, 10 mM Tris-HCl (pH 7.0), 1 mM EDTA, and 0.5% SDS) supplemented with 5% by volume proteinase K (20 mg/mL). RNA was isolated using TRIzol, followed by RT-qPCR analysis.

RNA immunoprecipitation (RIP)

RIP was performed as described previously[9]. Briefly, cells were lysed in polysome lysis buffer (100 mM KCl, 5 mM $MgCl_2$, 10 mM HEPES (pH 7.0), 0.5% NP-40, 1 mM DTT, and 2 mM vanadyl ribonucleoside complexes solution) supplemented with protease

inhibitor cocktail and RNase inhibitor, which were then subjected to immunoprecipitation (IP) with anti-ELAVL1 antibody (ABclonal, A19622) and washing with polysome lysis buffer for four times and polysome lysis buffer plus 1 M urea for four times. IP efficiency was detected by SDS-PAGE. RNAs were released by adding 150 μL of polysome lysis buffer with 0.1% SDS and 45 μg of proteinase K and incubated at 50°C for 30 min. RNA were extracted by Phenol-Chloroform-Isoamyl Alcohol mixture and followed by RT-qPCR analysis.

Xenograft tumor assay

For xenograft assay, female BALB/C nude mice (age 4-6 weeks) were subcutaneously implanted with 5×10^6 of sh-CTL, sh-circFOXK2-1, sh-circFOXK2-2-infected MCF7 cells suspended in PBS. For xenograft assays to assess the effects of ASO-circFOXK2, mice were subcutaneously implanted with 5×10^6 of MCF7 cells. After tumors were palpable, mice were randomized and administrated with ASO-CTL or ASO-circFOXK2 (5 nmol per dose, every 3 days) intratumorally for six cycles. For xenograft assays to assess the effects of ASO-circFOXK2 and tamoxifen, either individually or in combination, mice were subcutaneously implanted with 5×10^6 of MCF7 or TamR-MCF7 cells. After tumors were palpable, mice were randomized and administrated with ASO-CTL or ASO-circFOXK2 (2.5 nmol per dose, every 3 days) intratumorally for six cycles. Mice were challenged with vehicle or tamoxifen (20 mg/kg, every 3 days) intragastrically (i.g.) every other day for six cycles. Each nude mouse was brushed with estrogen (E_2, 10^{-2} M) every 3 days for the duration of the experiments to sustain xenograft tumor growth. Tumors were measured, both long

diameter (D) and short diameter (d), every 3 days with a caliper once palpable. Tumor volume was determined using the volume formula for an ellipsoid: $1/2 \times D \times d^2$. Mice were sacrificed when tumors reached 1,500 mm^3 or upon tumor ulceration/bleeding. Tumors were then excised, photographed, and weighted. Animals were housed in the Animal Facility at Xiamen University under pathogen-free conditions, following the protocol approved by the Xiamen Animal Care and Use Committee.

Immunohistochemistry (IHC)

Immunohistochemistry assay was performed following the protocol described previously[10]. Antibodies used are listed as following: Mouse monoclonal anti-Ki67 (Servicebio, GB121141-100, 1:300); Rabbit polyclonal anti-CCND1 (ABclonal, A11022, 1:100); Rabbit monoclonal anti-p-RB (Ser780) (ABclonal, AP0117, 1:50).

Statistics analysis

Gene expression data was expressed as the mean ± SD of at least three independent experiments. The comparison of two groups or data points was performed by using two-tailed Student's t-test. Multiple comparisons were analyzed by two-way analysis of variance (ANOVA). The p values below 0.05 were considered statistically significant (*$p < 0.05$, **$p < 0.01$, ***$p < 0.001$). Results from xenograft experiments and clinical breast samples were analyzed by GraphPad Prism 9.

References

1 Yi, J. *et al.* CircPVT1 promotes ER-positive breast tumorigenesis and drug resistance by targeting ESR1 and MAVS. *EMBO J* **42**, e112408, doi:10.15252/embj.2022112408 (2023).

2 Wang, L. *et al.* Estrogen-induced circRNA, circPGR, functions as a ceRNA to promote

estrogen receptor-positive breast cancer cell growth by regulating cell cycle-related genes. *Theranostics* **11**, 1732-1752, doi:10.7150/thno.45302 (2021).

3 Sherman, B. T. *et al.* DAVID: a web server for functional enrichment analysis and functional annotation of gene lists (2021 update). *Nucleic Acids Res* **50**, W216-W221, doi:10.1093/nar/gkac194 (2022).

4 Huang da, W., Sherman, B. T. & Lempicki, R. A. Systematic and integrative analysis of large gene lists using DAVID bioinformatics resources. *Nat Protoc* **4**, 44-57, doi:10.1038/nprot.2008.211 (2009).

5 Castellanos-Rubio, A. *et al.* A long noncoding RNA associated with susceptibility to celiac disease. *Science* **352**, 91-95, doi:10.1126/science.aad0467 (2016).

6 Gao, W. W. *et al.* Arginine methylation of HSP70 regulates retinoid acid-mediated RARbeta2 gene activation. *Proc Natl Acad Sci U S A* **112**, E3327-3336, doi:10.1073/pnas.1509658112 (2015).

7 Zhang, Y. *et al.* Analysis of the androgen receptor-regulated lncRNA landscape identifies a role for ARLNC1 in prostate cancer progression. *Nat Genet* **50**, 814-824, doi:10.1038/s41588-018-0120-1 (2018).

8 Chu, C., Quinn, J. & Chang, H. Y. Chromatin isolation by RNA purification (ChIRP). *J Vis Exp*, doi:10.3791/3912 (2012).

9 Peritz, T. *et al.* Immunoprecipitation of mRNA-protein complexes. *Nat Protoc* **1**, 577-580, doi:10.1038/nprot.2006.82 (2006).

10 Shen, H. F. *et al.* The Dual Function of KDM5C in Both Gene Transcriptional Activation and Repression Promotes Breast Cancer Cell Growth and Tumorigenesis. *Adv Sci* **8**, doi:10.1002/advs.202004635 (2021).

Supplementary figure legends

Figure S1. CircFOXK2 promotes ER-positive breast cancer cell growth.

(A, B) MCF7 cells transfected with control siRNA (si-CTL) or two independent siRNAs specifically targeting circFOXK2 (si-circFOXK2-1 and si-circFOXK2-2) were subjected to RT-qPCR (A) and immunoblotting (IB) (B) analysis (± SD, n.s: non-significant).

(C-E) T47D cells transfected with si-CTL, si-circFOXK2-1, or si-circFOXK2-2 were subjected to RT-qPCR (C), cell proliferation (D), and FACS (E) analysis (± SD, ***$P < 0.001$).

(F-H) T47D cells transfected with empty vector (EV) or vector expressing circFOXK2 were subjected to RT-qPCR (F), cell proliferation (G), and FACS (H) analysis (± SD, **$P < 0.01$, ***$P < 0.001$).

(I, J) MCF10A cells transfected with EV or vector expressing circFOXK2 were subjected to RT-qPCR (I) and cell proliferation (J) analysis (± SD, *$P < 0.05$, ***$P < 0.001$).

(K) The body weight of the mice as described in Figure 1I is shown.

(L, M) MCF7 cells infected with control shRNA (sh-CTL) or two independent shRNAs specifically targeting circFOXK2 (sh-circFOXK2-1 and sh-circFOXK2-2) were subjected to RT-qPCR (L) and IB (M) analysis (± SD, n.s: non-significant).

Figure S2. CircFOXK2 positively regulates the expression of CCND1 to activate E2F target genes and promote G1/S transition in the cell cycle.

(A-C) T47D cells transfected with control siRNA (si-CTL) or two independent siRNAs

specifically targeting circFOXK2 (si-circFOXK2-1 and si-circFOXK2-2) were subjected to RT-qPCR (A, C) and immunoblotting (IB) (B) analysis (± SD, *P < 0.05, **P < 0.01, ***P < 0.001).

(D-F) T47D cells transfected with si-CTL or si-circFOXK2 in the presence of empty vector (EV) or vector expressing circFOXK2 were subjected to RT-qPCR (D, F) and IB (E) analysis (± SD, *P < 0.05, **P < 0.01, ***P < 0.001).

Figure S3. CircFOXK2 recruits ELAVL1 to stabilize *CCND1* mRNA.

(A) ChIRP assay was performed by incubating cell lysates prepared from MCF7 cell with or without sense (*FOXK*-S) or anti-sense (*FOXK2*-AS) probe specifically targeting *FOXK2* followed by RT-qPCR analysis (± SD, n.s: non-significant, **P < 0.01).

(B-D) MCF7 cells transfected with si-CTL or siRNA specifically targeting FOXK2 (si-FOXK2) were subjected to IB (B), RT-qPCR (C), and cell proliferation (D) analysis (± SD, n.s: non-significant, *P < 0.05, **P < 0.01).

(E) MCF7 cells transfected with si-CTL or siRNA specifically targeting ELAVL1 (si-ELAVL1) were subjected to IB analysis.

(F, G) MCF7 cells were transfected with si-CTL or si-ELAVL1 and then treated with or without Actinomycin D (ActD, 10 μg/mL) for duration as indicated, followed by RT-qPCR analysis to examine the expression of *CCND1* (F) and *ACTIN* (G) (± SD, n.s: non-significant).

(H-J) The standard curves for calculating circFOXK2 (H), *CCND1* mRNA (I), and *FOXK2* mRNA (J) copy number.

(K) The copy numbers of circFOXK2, *CCND1* mRNA, and *FOXK2* mRNA in MCF7

cells are shown.

(L) Total RNAs extracted from MCF7 cells were incubated with or without RNase R (10 units/μg RNA) at 37°C for duration as indicated, followed by RT-qPCR analysis (± SD, n.s: non-significant, **P < 0.01, ***P < 0.001).

(M) MCF7 cells were treated with Actinomycin D (ActD, 10 μg/mL) for duration as indicated, followed by RT-qPCR analysis (± SD, n.s: non-significant, ***P < 0.001).

Figure S4. ASO-circFOXK2 suppresses ER-positive breast cancer cell growth both *in vitro* and *in vivo*.

(A, D-G) T47D cells transfected with control ASO (ASO-CTL) or ASO specifically targeting circFOXK2 (ASO-circFOXK2) were subjected to RT-qPCR (A, E), IB (D), FACS (F), and cell proliferation (G) analysis (± SD, *P < 0.05, **P < 0.01, ***P < 0.001).

(B, C) MCF7 cells transfected with ASO-CTL or ASO-circFOXK2 were subjected to RT-qPCR (B) and IB (C) analysis (± SD, n.s: non-significant).

(H) The body weight of the mice as described Figure 4F is shown.

Figure S5. Combination treatment with ASO-circFOXK2 and tamoxifen shows synergistic effects in suppressing ER-positive breast cancer cell growth both *in vitro* and *in vivo*.

(A-C) T47D cells were transfected with ASO-CTL or ASO-circFOXK2 for 36 h before treating with or without tamoxifen (Tam, 5 μM) for 36 h, followed by IB (A), RT-qPCR (B), and FACS (C) analysis (± SD, *P < 0.05, **P < 0.01, ***P < 0.001).

(D) T47D cells were transfected with ASO-CTL or ASO-circFOXK2 and treated with

or without tamoxifen (Tam, 5 μM), followed by cell proliferation assay (± SD, *P < 0.05, **P < 0.01, ***P < 0.001).

(E) The body weight of the mice as described in Figure 5E is shown.

Figure S6. ASO-circFOXK2 re-sensitizes tamoxifen-resistant ER-positive breast cancer cells to tamoxifen treatment.

(A, B) TamR-MCF7 cells were treated with or without tamoxifen (Tam, 5 μM) for duration as indicated, followed by RT-qPCR (A) and IB (B) analysis (± SD, n.s: non-significant).

(C) TamR-MCF7 cells transfected with control siRNA (si-CTL) or siRNA specifically targeting CCND1 (si-CCND1) for 36 h before treating with or without tamoxifen (Tam, 5 μM) for 36 h, followed by FACS analysis (± SD, n.s: non-significant, *P < 0.05, ***P < 0.001).

(D) TamR-MCF7 cells were transfected with si-CTL or si-CCND1 and treated with or without tamoxifen (Tam, 5 μM), followed by cell proliferation assay (± SD, n.s: non-significant, *P < 0.05, **P < 0.01)

(E) The body weight of the mice as described in Figure 6E is shown.

Supplementary table legends

Table S1. The list of circFOXK2-regulated genes, along with the gene ontology analysis results for these genes, is provided.

Table S2. The sequence information for the RT-qPCR primers utilized in the current study is provided. The sequence information for the RT-qPCR primers designed to detect the expression of mRNAs, circRNAs, snoRNAs, and lncRNAs as

indicated is provided. F: forward; R: reverse.

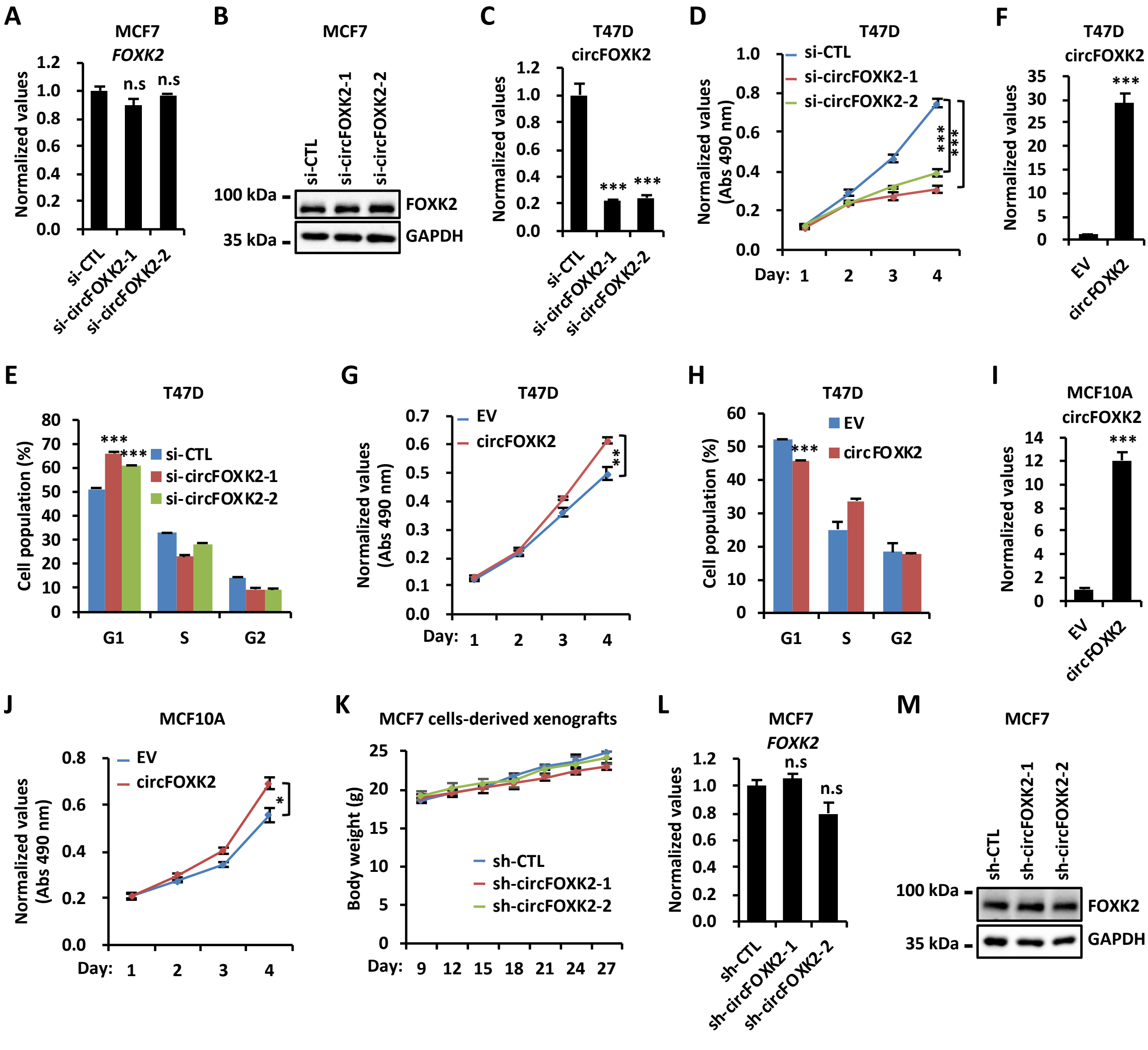
A
MCF7
FOXK2
Normalized values
n.s
n.s
si-CTL
si-circFOXK2-1
si-circFOXK2-2
B
MCF7
si-CTL
si-circFOXK2-1
si-circFOXK2-2
100 kDa
35 kDa
FOXK2
GAPDH
C
T47D
circFOXK2
Normalized values
***
***
D
T47D
si-CTL
si-circFOXK2-1
si-circFOXK2-2
Normalized values (Abs 490 nm)
Day:
F
T47D
circFOXK2
Normalized values
EV
circFOXK2
E
T47D
Cell population (%)
si-CTL
si-circFOXK2-1
si-circFOXK2-2
G1
S
G2
G
T47D
EV
circFOXK2
Normalized values (Abs 490 nm)
Day:
H
T47D
Cell population (%)
EV
circFOXK2
I
MCF10A
circFOXK2
Normalized values
J
MCF10A
EV
circFOXK2
Normalized values (Abs 490 nm)
Day:
K
MCF7 cells-derived xenografts
Body weight (g)
sh-CTL
sh-circFOXK2-1
sh-circFOXK2-2
Day:
L
MCF7
FOXK2
Normalized values
sh-CTL
sh-circFOXK2-1
sh-circFOXK2-2
M
MCF7
sh-CTL
sh-circFOXK2-1
sh-circFOXK2-2
100 kDa
35 kDa
FOXK2
GAPDH

Figure S1

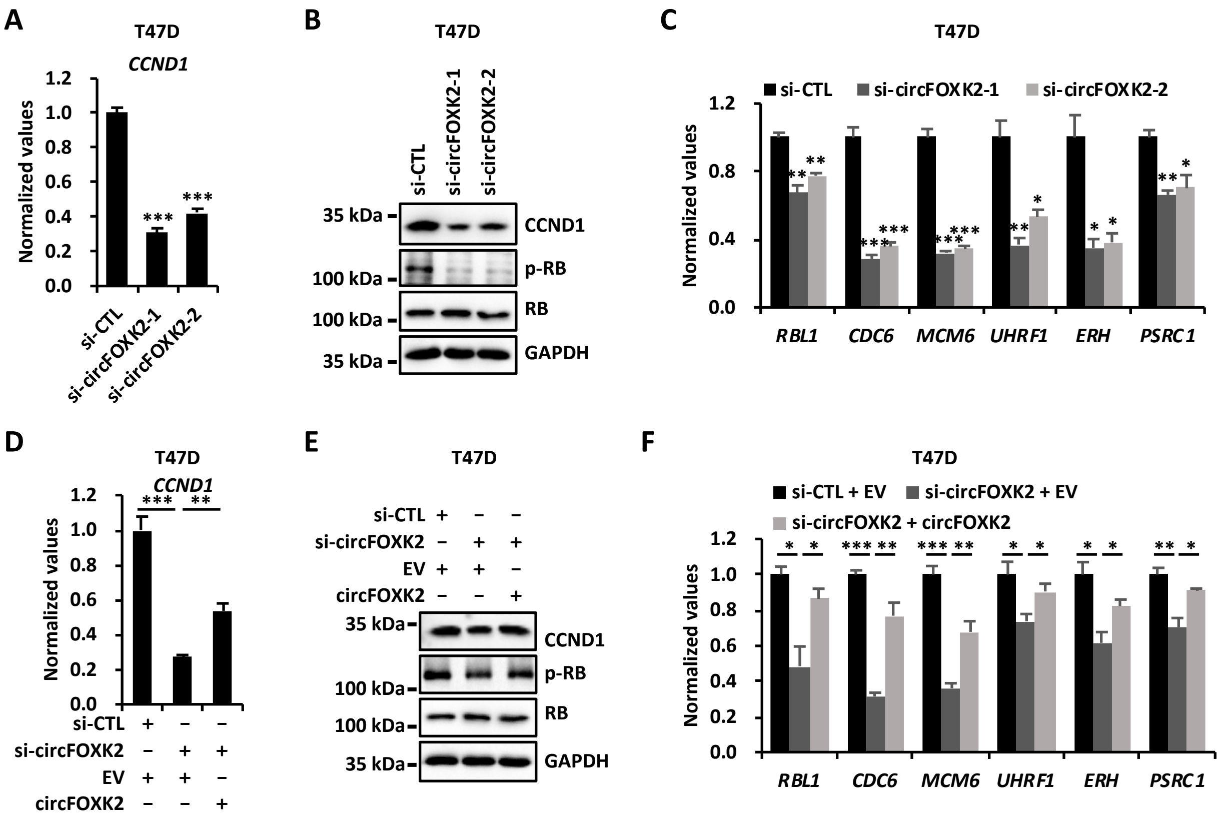
A
T47D
CCND1
Normalized values
si-CTL
si-circFOXK2-1
si-circFOXK2-2
B
T47D
si-CTL
si-circFOXK2-1
si-circFOXK2-2
35 kDa
100 kDa
100 kDa
35 kDa
CCND1
p-RB
RB
GAPDH
C
T47D
si-CTL
si-circFOXK2-1
si-circFOXK2-2
Normalized values
RBL1
CDC6
MCM6
UHRF1
ERH
PSRC1
D
T47D
CCND1
Normalized values
si-CTL
si-circFOXK2
EV
circFOXK2
E
T47D
si-CTL
si-circFOXK2
EV
circFOXK2
CCND1
p-RB
RB
GAPDH
F
T47D
si-CTL + EV
si-circFOXK2 + EV
si-circFOXK2 + circFOXK2
Normalized values
RBL1
CDC6
MCM6
UHRF1
ERH
PSRC1

Figure S2

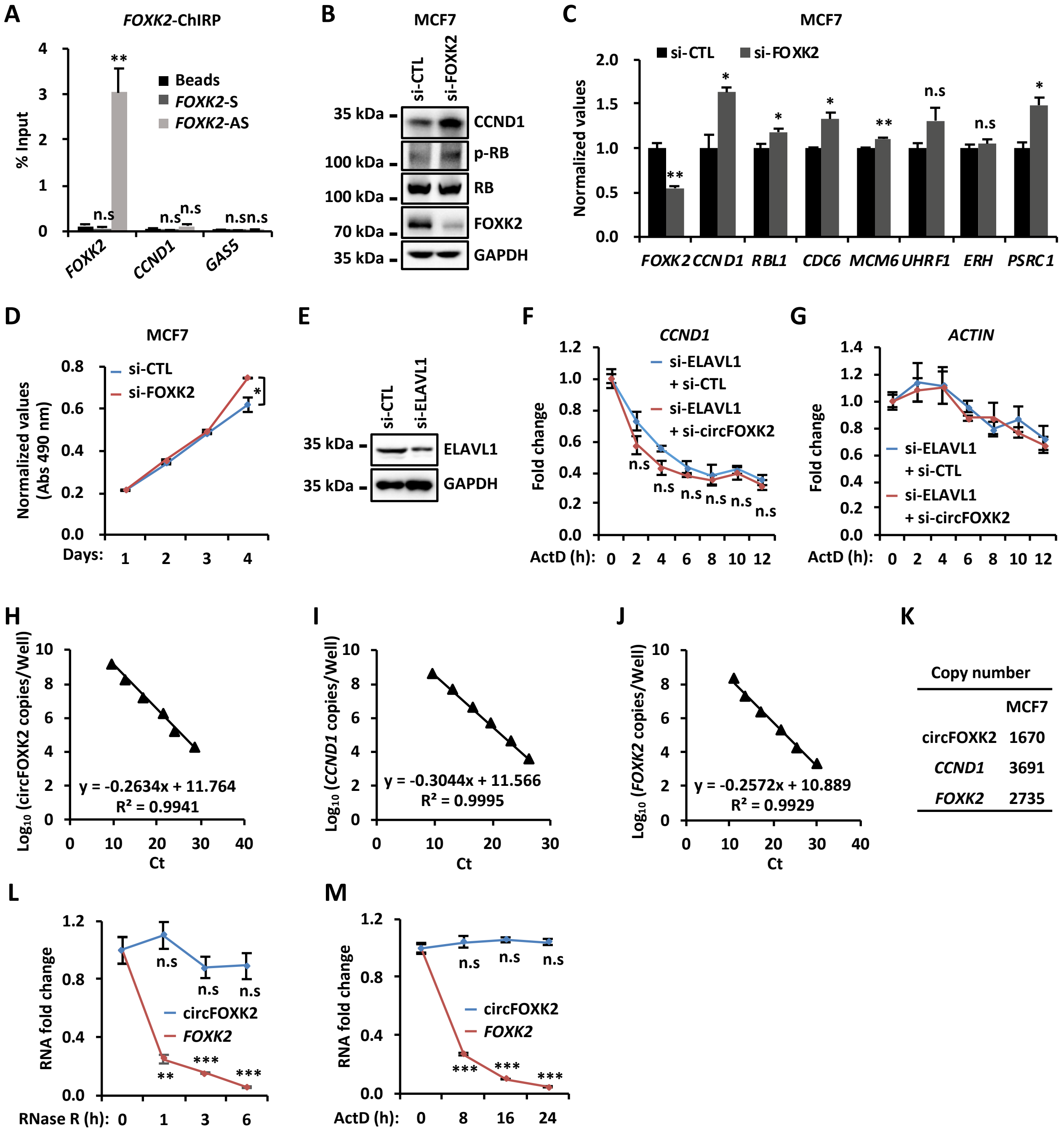

Copy number	
	MCF7
circFOXK2	1670
CCND1	3691
FOXK2	2735

Figure S3

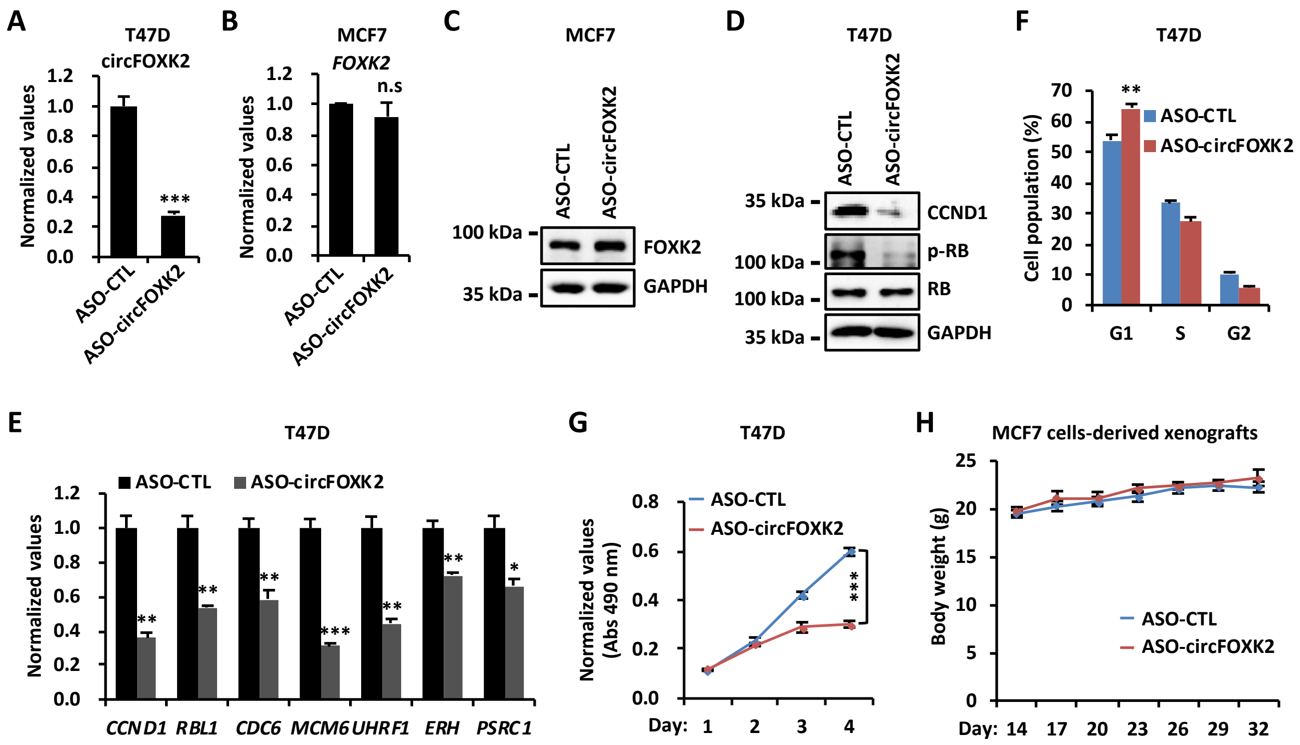
A
T47D
circFOXK2
Normalized values
ASO-CTL
ASO-circFOXK2
***
B
MCF7
FOXK2
n.s
C
MCF7
100 kDa
35 kDa
FOXK2
GAPDH
D
T47D
CCND1
p-RB
RB
F
Cell population (%)
G1
S
G2
**
E
CCND1
RBL1
CDC6
MCM6
UHRF1
ERH
PSRC1
G
Normalized values (Abs 490 nm)
Day:
H
MCF7 cells-derived xenografts
Body weight (g)

Figure S4

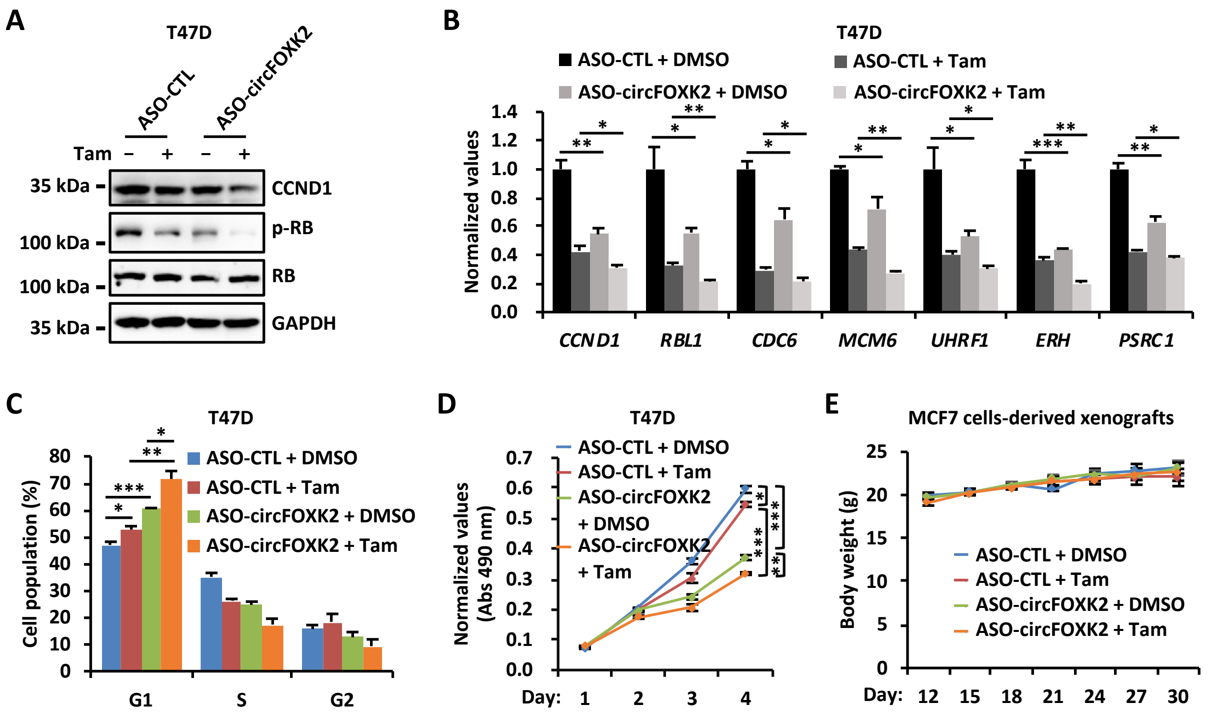
A
T47D
ASO-CTL
ASO-circFOXK2
Tam
35 kDa
CCND1
p-RB
100 kDa
RB
100 kDa
35 kDa
GAPDH
B
T47D
ASO-CTL + DMSO
ASO-CTL + Tam
ASO-circFOXK2 + DMSO
ASO-circFOXK2 + Tam
Normalized values
CCND1
RBL1
CDC6
MCM6
UHRF1
ERH
PSRC1
C
T47D
Cell population (%)
G1
S
G2
D
T47D
Normalized values (Abs 490 nm)
Day:
E
MCF7 cells-derived xenografts
Body weight (g)
Day:

Figure S5

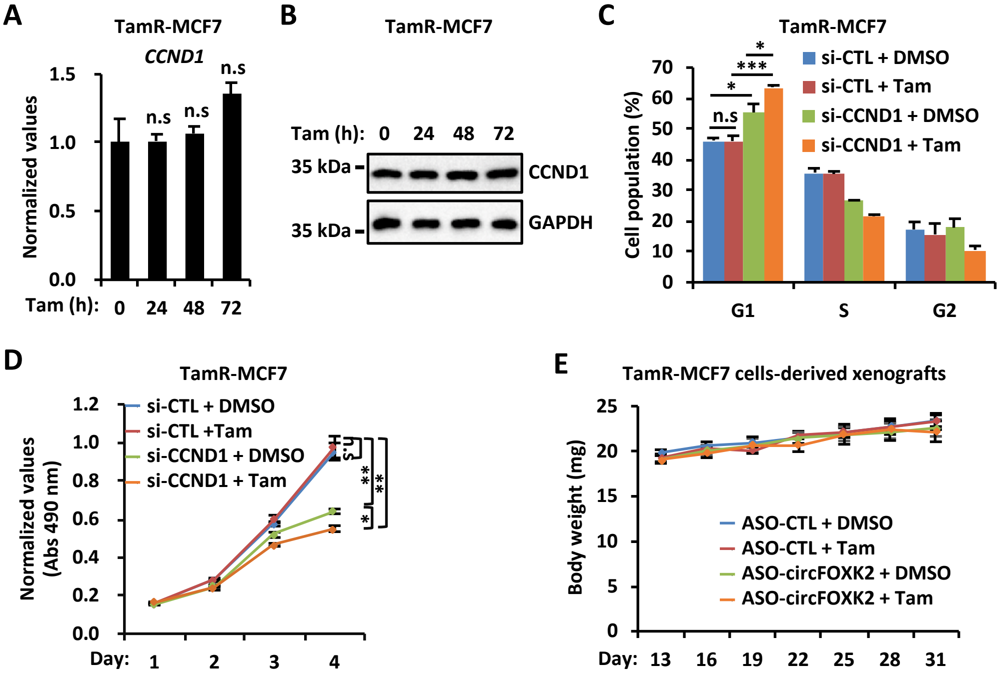
A
TamR-MCF7
CCND1
Normalized values
1.5
1.0
0.5
0.0
n.s
n.s
n.s
Tam (h): 0 24 48 72
B
TamR-MCF7
Tam (h): 0 24 48 72
35 kDa
CCND1
35 kDa
GAPDH
C
TamR-MCF7
Cell population (%)
70
60
50
40
30
20
10
0
si-CTL + DMSO
si-CTL + Tam
si-CCND1 + DMSO
si-CCND1 + Tam
n.s
*
***
*
G1
S
G2
D
TamR-MCF7
Normalized values (Abs 490 nm)
1.2
1.0
0.8
0.6
0.4
0.2
0.0
si-CTL + DMSO
si-CTL +Tam
si-CCND1 + DMSO
si-CCND1 + Tam
n.s
**
**
*
Day: 1 2 3 4
E
TamR-MCF7 cells-derived xenografts
Body weight (mg)
25
20
15
10
5
0
ASO-CTL + DMSO
ASO-CTL + Tam
ASO-circFOXK2 + DMSO
ASO-circFOXK2 + Tam
Day: 13 16 19 22 25 28 31

Figure S6
